# Vessel‐On‐A‐Chip Coupled Proteomics Reveal Pressure‐Overload‐Induced Vascular Remodeling

**DOI:** 10.1002/advs.202415024

**Published:** 2025-03-24

**Authors:** Yanjun Liu, Jianxujie Zheng, Lingyan Zhong, Zengyu Wang, Dan Zhao, Hong Lin, Xiaoxue Zhang, Ke Meng, Xiaoxia Yang, Dongxue Zhang, Ling Lin, Liang Qiao

**Affiliations:** ^1^ Department of Chemistry Zhongshan Hospital Fudan University Shanghai 200000 China; ^2^ Department of Neurosurgery Zhongshan Hospital (Xiamen) Fudan University Xiamen Fujian 361015 China

**Keywords:** hypertension, microfluidics, proteomics, smooth muscle cells, vessel‐on‐a‐chip

## Abstract

Hypertension, the leading cause of cardiovascular disease and premature mortality, is characterized by increased vessel stretch and alterations in vascular smooth muscle cells (VSMCs). In this study, a vessel‐on‐a‐chip model is developed to simulate both physiological and pathological stretch conditions alongside a mouse model of hypertension. Proteomics analysis is applied to investigate changes in VSMCs using the vessel‐on‐a‐chip system and compared these findings with data from the mouse model. The results demonstrates that physiological stretch enhances the expression of contractile markers in VSMCs. Additionally, the chip effectively replicates cellular responses to pathological stretch and stress, including the upregulation of ERK signaling, calcium ion transport pathway, integrin signaling pathway, endoplasmic reticulum stress, toll‐like receptor activation, oxidative stress, and synthesis of sphingolipids and ceramides. These findings indicate that the vessel chip successfully mimics in vivo biological events associated with hypertension. The vessel‐on‐a‐chip system holds promise for advancing the study of vessel‐related diseases and facilitating the development of novel hypertension therapeutics.

## Graphical abstract

A vessel‐on‐a‐chip system is developed to replicate both physiological and pathological vessel cyclic stretches. Proteomic analysis reveals pressure‐overload‐induced vascular remodeling, including cellular response to mechanical cues, cellular oxidative stresses, ER stress, and ceramide biosynthesis activation in the vessel‐on‐a‐chip system, well aligned with the hypertensive mouse model by Ang II infusion.

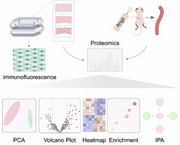

## 1 Introduction

Hypertension is the primary contributor to cardiovascular diseases (CVDs) and a leading factor in premature mortality worldwide, associated with vascular changes characterized by vascular remodeling.^[^ ^1^, ^2^, ^3^ ^]^ Vascular smooth muscle cells (VSMCs) comprise the medial layer of the vessel wall, playing a critical role in maintaining vascular integrity and responding to pathological changes.^[^ ^4^ ^]^ In their quiescent state within the medial layer, VSMCs are largely inactive and maintain a highly differentiated contractile phenotype. However, following vascular injury, VSMCs undergo phenotypic transformation, acquiring proliferative, secretory, and migratory properties. In hypertension, VSMCs dedifferentiate, losing their contractile phenotype and exhibiting increased proliferation, migration, and inflammation.^[^ ^2^ ^]^

2D cell culture models are widely employed in vitro to study VSMCs changes associated with CVDs. Wang et al.^[^ ^5^ ^]^ demonstrated that in vitro knockdown of the orphan receptor GPRC5C in human aortic VSMCs reduced angiotensin II (Ang II)‐induced inositol phosphate production and myosin light chain phosphorylation, thereby decreasing Ang II binding to AT1 receptors and alleviating established arterial hypertension. These cell models offer a simplified system that allows for controlled experimental conditions. However, such models diverge significantly from living organism, lacking essential in vivo environmental features such as mechanical stretch and fluid shear forces.^[^ ^6^ ^]^

Animal models are also widely utilized in CVDs research, with Ang II‐treated mice being particularly common for investigating pressure overload‐induced remodeling and related pathologies.^[^ ^7^, ^8^ ^]^ Using an Ang II‐treated mouse model, Wu et al.^[^ ^8^ ^]^ identified cytosolic citrate accumulation within VSMCs during Ang II‐induced vascular remodeling in mice, promoting the transformation of VSMCs toward a pro‐inflammatory phenotype. However, animal models are constrained by species differences and ethical concerns, and often require prolonged periods to observe disease progression. Furthermore, it is estimated that only ≈20% of CVDs drugs effective in animal models demonstrate therapeutic efficacy in humans.^[^ ^9^ ^]^ This limitation underscores the challenges of relying on animal models for studying human diseases.

Traditional mechanistic studies predominantly rely on in vitro cell experiments and animal models, both of which insufficiently replicate human physiological characteristics. Moreover, clinical sample analyses lack precise control over individual pathogenic factors, highlighting the urgent need for a novel technological platform to bridge in vitro models, animal studies, and clinical samples. Such a platform would facilitate the deconstruction and biomimicry of in vivo microenvironments, enabling the stepwise isolation of potential pathogenic factors critical to understanding vascular remodeling mechanisms. Advanced platforms, such as organ‐on‐a‐chip models and organoids, have been proposed to meet these needs in CVDs research.

Organ‐on‐a‐chip systems integrate microengineering and microfluidic technologies to recreate cellular environments and biological events in vivo, including the simulation of biophysical forces.^[^ ^10^ ^]^ Meijer et al.^[^ ^11^ ^]^ constructed a vessel chip using gelatin methacryloyl to replicate the 3D substrate of VSMCs in vivo, highlighting the critical role of the microenvironment in regulating VSMCs behavior. Their findings demonstrated that VSMCs cultured on gelatin methacryloyl substrates with varied functionalization and Young's modulus were inclined toward a contractile phenotype. Chen et al.^[^ ^12^ ^]^ developed a vessel chip to investigate the role of mechanical cues in VSMC phenotype switching. Cyclic stretch at 1 Hz enhanced the contractile phenotype of VSMCs, whereas higher frequencies induced inflammation, underscoring the impact of mechanical stretch. However, these studies primarily elucidate phenotype switching in VSMCs, and a more comprehensive understanding of VSMCs dynamics during disease progression is needed. Integrating these models with advanced analytical methodologies could help bridge this knowledge gap.

Proteins are essential functional molecules in living organisms, making their direct characterization vital to understanding biological functions. Liquid chromatography‐tandem mass spectrometry (LC‐MS/MS)‐based proteomics enables comprehensive protein profiling across cells, tissues, and organisms under specific conditions, encompassing protein identification, quantification, and post‐translational modifications.^[^ ^13^ ^]^ Recently, with the advances of mass spectrometry techniques and artificial intelligence,^[^ ^14^, ^15^ ^]^ proteomic analysis from a low amount of sample is possible, further advancing personalized risk stratification, early diagnosis, and precision therapy.^[^ ^16^, ^17^ ^]^ Therefore, integrating advanced organ‐on‐a‐chip systems with cutting‐edge proteomics techniques is feasible and essential for gaining a comprehensive understanding of disease mechanisms, such as vascular remodeling in hypertension.

In this study, we developed a vessel‐on‐a‐chip system comprising a cell culture layer and a pneumatic layer separated by a polydimethylsiloxane (PDMS) membrane. By applying varying cyclic pressures to the pneumatic layer, the chip can simulate both physiological and hypertensive vascular conditions. Proteomic analysis was conducted to characterize protein and pathway changes in VSMCs under these conditions. Additionally, an in vivo mouse model of hypertension was constructed to enable a comparative analysis with the in vitro vessel chip model. Our results demonstrated that the vessel chip effectively replicates several hypertension‐associated characteristics in VSMCs, including their responses to mechanical cues and cellular stress. This approach offers significant potential for advancing research into vessel‐related diseases and facilitating the development of novel therapeutic strategies.

## 2 Results and Discussion

### 2.1 The Vessel‐On‐A‐Chip System

**Figure 1:**
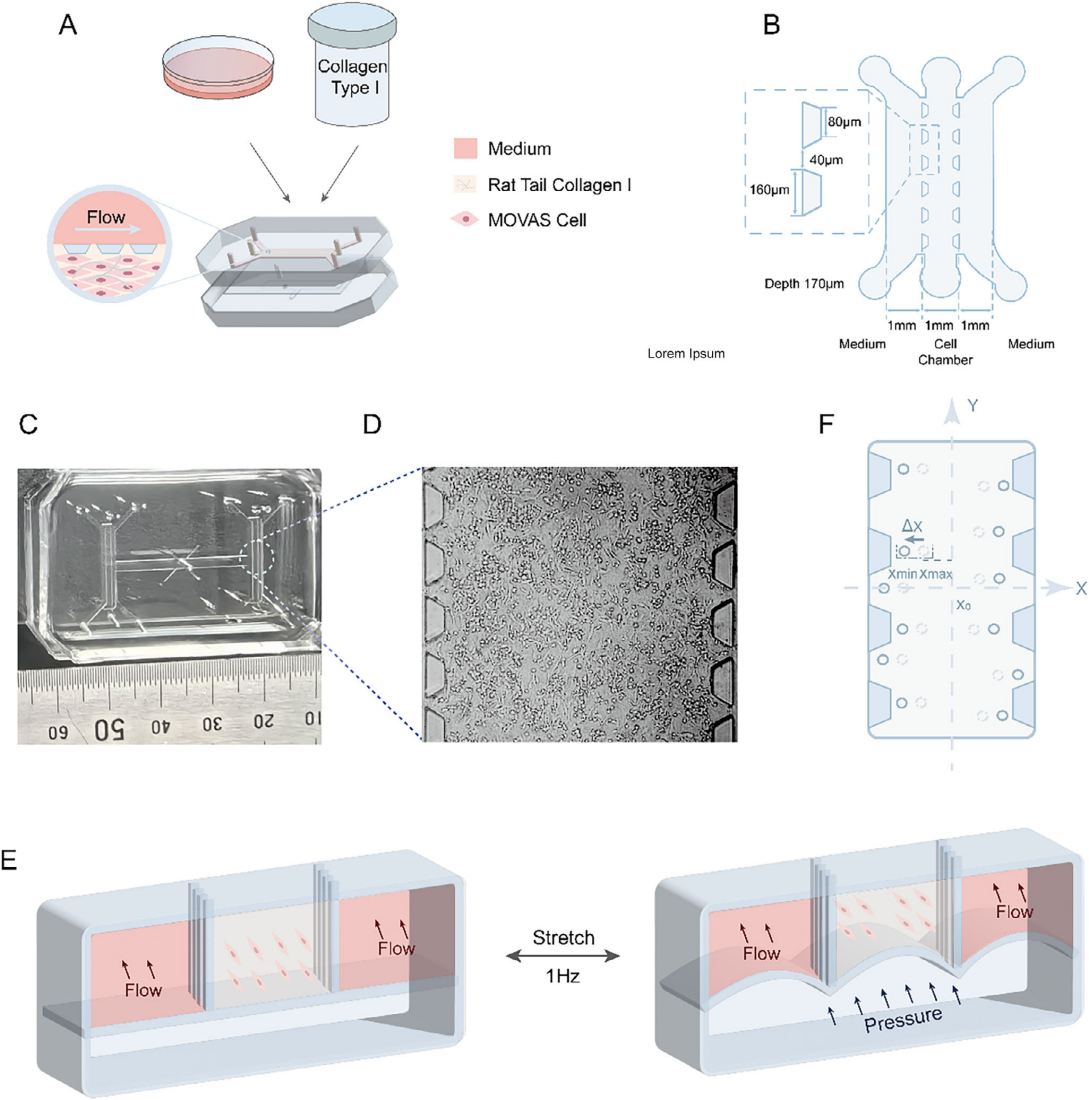
Illustration of the vessel‐on‐a‐chip system. A,B) Structure of the vessel chip. The chip consisted of a cell culture layer and a pneumatic layer separated by a PDMS membrane. The upper cell culture layer contained two columns of trapezoidal pillars to separate the layer into cell culture channel and flanking medium channels. A mixture of rat tail collagen type I and the mouse aortic smooth muscle cell line MOVAS was injected into the middle channel and polymerized, followed by introducing culture medium into the two flanking medium channels. C) Image of the vessel chip viewed from above. D) Cells cultured in the vessel chip. E) Deformation of the cell‐containing collagen during cyclic stretch. F) Measurement of the deformation of the collagen using polystyrene microspheres.

To mimic the in vivo arterial environment, we constructed a vessel‐on‐a‐chip system. The device consists of a cell culture layer and a pneumatic layer separated by an elastic membrane (**Figure** **1**A–C). PDMS was selected for fabricating the microfluidic chip due to its transparency and biocompatibility. Additionally, PDMS's elasticity allows it to deform under pressure. The upper layer includes two columns of trapezoidal pillars, dividing it into a central cell culture channel and two flanking medium channels, each 1 mm in width. The gap between adjacent pillars is 40 µm. Although the central channel is not fully enclosed, the pillars effectively confine liquid within this channel due to surface tension. A pre‐polymer mixture of rat tail collagen and the mouse aortic smooth muscle cell line (MOVAS) was introduced into the cell culture channel. After 15 min of polymerization, the cells were encapsulated within the collagen structure (Figure 1D).

To induce cyclic stretch, the bottom layer of the device was connected to a push‐pull system that provided periodic pressure, with the frequency set at 1 Hz. During each cycle, pressure application deformed the PDMS membrane, compressing the collagen‐encapsulated cells; upon release, both the membrane and collagen returned to their original shapes (Figure 1E; Video S1, Supporting Information). To quantify deformation, polystyrene microspheres were mixed with rat tail collagen and injected into the channel, where their displacement reflected collagen stretching (Figure 1F; Video S2, Supporting Information). Deformation was defined as the ratio of the distance traveled by a microsphere in one cycle to its minimum distance from the channel center. Significant displacement can be observed in the x‐axis. There was little displacement in the y‐axis (only ≈0.9% to the total displacement of the beads in x‐ and y‐ axis). There should also be significant displacement in the z‐axis based on the design of the microfluidic chip, which, however, cannot be measured using the microscopic characterization system.

In normal aortic and other elastic arteries, vessels expand by ≈10% with each heartbeat.^[^ ^18^ ^]^ By contrast, hypertensive vessels are exposed to increased cyclic stretch.^[^ ^19^ ^]^ In vitro models typically classify 5–10% cyclic stretch as physiological and cyclic stretch exceeding 15% as pathological.^[^ ^20^ ^]^ Accordingly, we applied cyclic stretch to simulate both physiological and pathological conditions. When a pressure range of 0 to 30 mbar was applied, the average x‐axis deformation measured 7.6 ± 4.1% based on a total of 450 microspheres from the left and right sides randomly selected from three independent chips (150 microspheres per chip). The three chips showed highly reproducible results, where the average ± standard deviation of the three chips based on their respective average x‐axis deformation was 7.6 ± 0.2%. Increasing the pressure range to 0–110 mbar resulted in an average x‐axis deformation of 19.0 ± 9.3% and a reproducibility evaluation of 19.0 ± 1.0% across three chips. These deformation values closely correspond to physiological and pathological vessel expansion, respectively. Thus, we selected 30 mbar and 110 mbar as cyclic pressures to model physiological and pathological vessel stretching, respectively.

Notably, the extracellular matrix (ECM) exhibits nonelastic properties, yet mechanical forces can induce ECM remodeling.^[^ ^21^, ^22^ ^]^ Under high strain, collagen may reorganize, potentially modifying mechanical signaling and influencing cell function. To assess the stability of the microfluidic system over time, we measured collagen deformation at 0h (7.6 ± 4.1% for 30 mbar, 19.0 ± 9.3% for 110 mbar) and 24 h (7.3 ± 3.5% for 30 mbar, 18.3 ± 13.2% for 110 mbar). No significant changes were observed under either condition (Figure S1, Supporting Information), indicating that cells experienced a consistent mechanical environment and pressure throughout the 24‐h period.

### 2.2 Physiological Stretch Promotes the Contractile Phenotype of MOVAS

**Figure 2:**
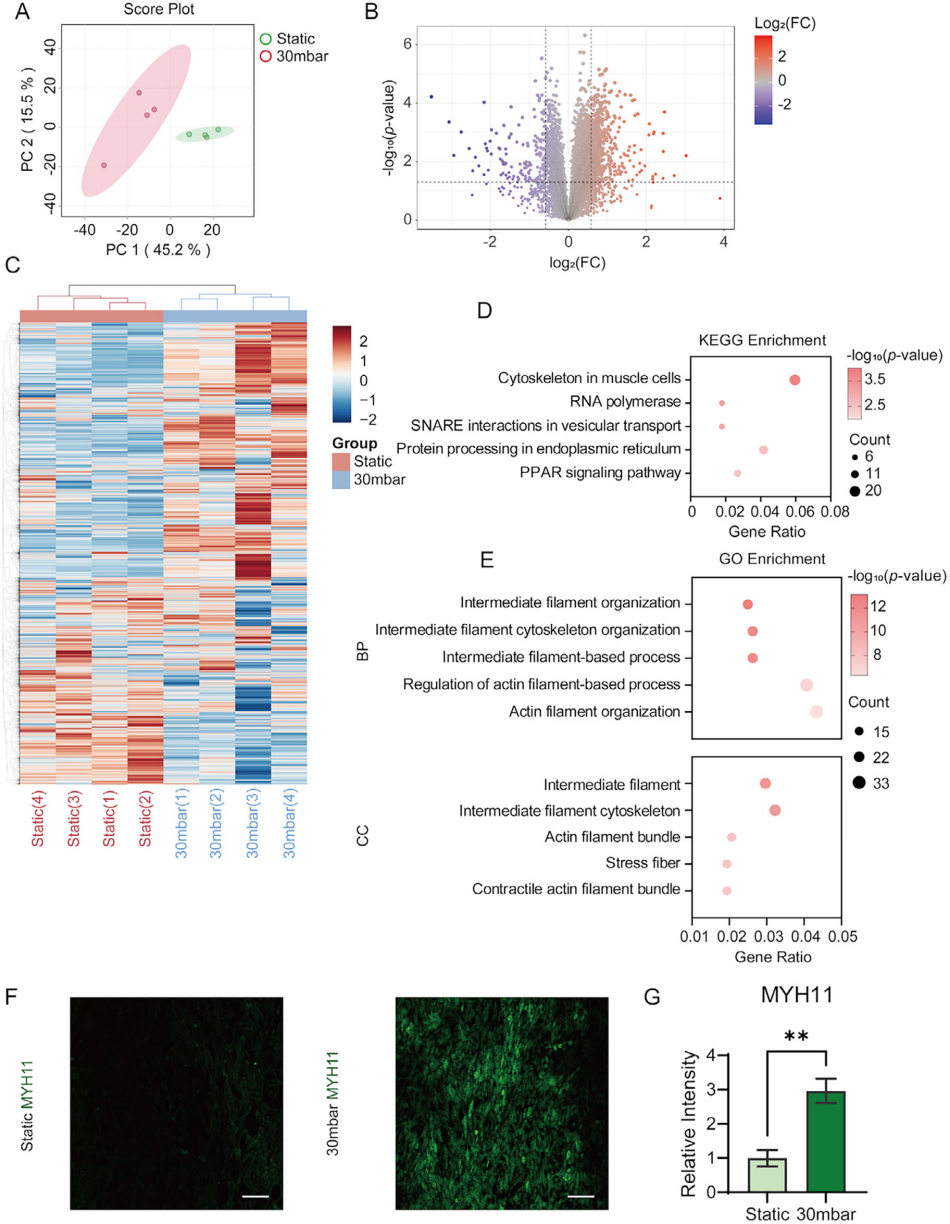
Proteomic and immunofluorescence characterization of the MOVAS cells under physiological stretch and static culture. A) PCA score plots for proteomic data comparing physiologically stretched and static cultured cells. B) Volcano plots displaying identified proteins in MOVAS with log_2_FC as the horizontal axis and −log_10_ *p*‐value as the vertical axis. The thresholds were defined as 0.05 for the *p*‐value and 0.585 for |log_2_FC|. FC, fold change in protein expression of the physiologically stretched group compared to the static group. C) Heatmap clustering of physiological stretched and static group cells based on the z‐scores for protein contents. Pearson correlation was used as the distance measurement for both the columns and rows. D,E) KEGG and GO enrichment analysis of the significantly changed proteins obtained from (C). BP, Biological Process; CC, Cellular Component. F) Fluorescence images of the static and physiological stretched groups stained for MYH11. The scale bar indicates 100 µm. G) Quantification of MYH11 fluorescence intensity. Data presented as mean±standard deviation, n = 3, *p*‐values were calculated using two‐tailed *t*‐test. ^**^ *p* < 0.01.

Untargeted proteomic analysis was performed on MOVAS cells in both static conditions and under physiological cyclic stretch (30 mbar) after 24 h. A total of 9083 proteins (Table S1, Supporting Information) were identified and quantified across the two groups. Principal component analysis (PCA) clearly separated the static and physiological stretch groups, indicating a significant difference between them (**Figure** **2**A). Proteins that exhibited significant changes were identified based on criteria of *p*‐value < 0.05 and fold change (FC), resulting in 669 upregulated (FC >1.5) and 161 downregulated (FC< 2/3) proteins (Figure 2B; Table S2, Supporting Information). Heatmap analysis of all identified proteins further showed distinct clustering between the two groups (Figure 2C).

Among the upregulation proteins, several associated with VSMC phenotype regulation and inflammation were identified, including myosin regulatory light polypeptide 9 (MYL9), four and a half LIM domains protein 2 (FHL2), and tropomyosin alpha‐1 chain (TPM1) (Figure S2, Supporting Information). MYL9 and TPM1 are positively correlated with the maintenance of the VSMC contractile phenotype,^[^ ^23^, ^24^ ^]^ while FHL2 is linked to inflammation suppression.^[^ ^25^ ^]^ The observed upregulation of these proteins suggested that physiological stretch enhanced the contractile phenotype of the MOVAS cells. KEGG pathway enrichment analysis and Gene Ontology (GO) enrichment analysis revealed notable enrichment of pathways related to the cell cytoskeleton (Figure 2D,E). The cytoskeleton, which includes intermediate filaments, actin, and other components, is crucial for the contractile function and phenotype of VSMCs.^[^ ^26^ ^]^ Intermediate filaments play a significant role in the development of smooth muscle force. Upon activation, they undergo spatial rearrangement that is associated with cytoskeletal reorganization.^[^ ^27^ ^]^ Actin also contributes to cytoskeletal dynamics, which is essential for the contraction of smooth muscle tissues.^[^ ^28^ ^]^

In healthy arteries, VSMCs typically adopt a differentiated, contractile phenotype, characterized by high expression of smooth muscle myosin heavy chain 11 (MYH11).^[^ ^29^ ^]^ We employed immunofluorescence to quantify MYH11 expression in VSMCs within our microfluidic chips. The physiological stretch group showed significantly higher fluorescence intensity compared to the static group (Figure 2F,G), indicating a healthier state of MOVAS cells under physiological stretching. These results collectively demonstrate the successful establishment of a vessel‐on‐a‐chip system that recapitulates physiological conditions.

### 2.3 Proteomics Uncovers Dysregulation of MOVAS Under Pathological Stretch

**Figure 3:**
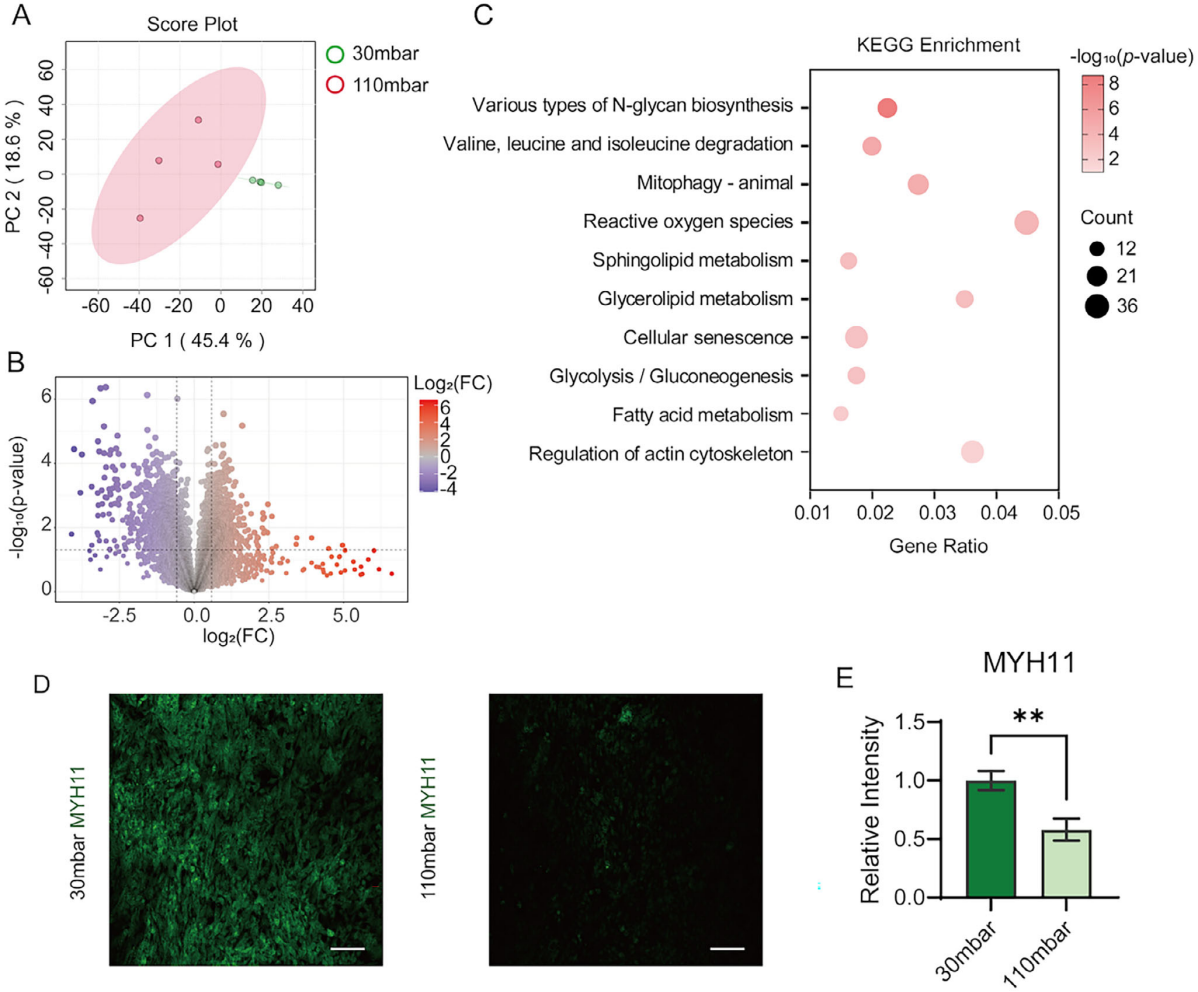
Proteomic and immunofluorescence characterization of the MOVAS cells under physiological and pathological stretches. A) PCA score plots for proteomic data comparing cells cultured under pathological and physiological stretches. B) Volcano plots displaying identified proteins in MOVAS with log_2_FC as the horizontal axis and −log_10_ *p*‐value as the vertical axis. The thresholds were set as 0.05 for the *p*‐value and 0.585 for |log_2_FC|. FC, fold change in protein expression of the pathological stretched group compared to the physiological group. C) Pathways from KEGG enrichment analysis of the significantly changed proteins obtained from (B). D) Fluorescence images of MYH11 staining in the physiological and pathological stretched groups after two days of culture. The scale bar indicates 100 µm. E) Quantification of MYH11 fluorescence intensity. Data presented as mean±standard deviation, n = 3, *p*‐values were calculated using two‐tailed *t*‐test. ^**^ *p* < 0.01.

We conducted untargeted proteomics on MOVAS cells subjected to both physiological (30 mbar) and pathological (110 mbar) stretches. In total, 9102 proteins (Table S3, Supporting Information) were identified and quantified across both groups. PCA revealed a clear separation between the physiological and pathological stretch groups, indicating significant changes in MOVAS cells under pathological stretch (**Figure** **3**A). Heatmap analysis of all identified proteins further showed distinct clustering between the two groups (Figure S3, Supporting Information). Proteins that exhibited significant changes were identified using the criteria of *p*‐value < 0.05 and FC, resulting in 829 upregulated (FC > 1.5) and 952 downregulated (FC < 2/3) proteins (Figure 3B; Table S4, Supporting Information). KEGG pathway enrichment analysis indicated that pathological stretch impacted multiple pathways (Figure 3C), notably those regulating the actin cytoskeleton, reactive oxygen species, sphingolipid metabolism, glycolysis and fatty acid metabolism. Mechanical stress triggers the cytoskeleton remodeling through various signaling pathways.^[^ ^30^, ^31^ ^]^ The enrichment of actin cytoskeleton regulation reflected a cellular response to pathological stretch. Additionally, mechanical cues can affect a range of cellular functions, including morphology, differentiation, proliferation, adhesion, growth, and migration.^[^ ^32^ ^]^ The enrichment of pathways related to reactive oxygen species and sphingolipid metabolism suggested a heightened cellular stress response.^[^ ^33^, ^34^ ^]^ Moreover, the enrichment of glycolysis and fatty acid metabolism pathways implied metabolic reprogramming in MOVAS, which is crucial for phenotypic switching.^[^ ^35^ ^]^ In hypertension and other vascular disorders, the contractile phenotype of VSMCs is compromised, typically reflected by decreased expression of the contractile marker MYH11.^[^ ^29^, ^36^ ^]^ Immunofluorescence analysis confirmed this in MOVAS cells (Figure 3D,E), showing reduced MYH11 expression under pathological stretch and indicating loss of the contractile phenotype.

To corroborate the vessel chip findings, we established a hypertensive mouse model using Ang II (Figure S4A, Supporting Information). Ang II administration via mini‐osmotic pumps significantly elevated both systolic and diastolic blood pressure relative to saline‐injected controls (Figure S4B, Supporting Information). Vessels harvested following Ang II infusion displayed marked collagen deposition (Figure S4C,D, Supporting Information). Quantitative analysis of trichrome staining images showed that the collagen area ratio significantly increased in the Ang II infusion group, indicating vascular remodeling (Figure S4E, Supporting Information). After 14 days of Ang II treatment, proteomic analysis of the mouse aortas identified 8234 proteins, with 751 upregulated and 612 downregulated in the Ang II‐treated group compared to the controls (Figure S5A and Tables S5 and S6, Supporting Information). PCA clearly distinguished the Ang II‐treated group from saline‐injected controls (Figure S5B, Supporting Information), which was further confirmed by heatmap clustering (Figure S5C, Supporting Information). KEGG enrichment analysis of the dysregulated proteins indicated that hypertensive mouse vessels also responded to pathological mechanical signals and cellular stress, along with undergoing metabolic reprogramming (Figure S5D, Supporting Information).

### 2.4 Proteomics Reveals MOVAS Response to Mechanical Cues

**Figure 4:**
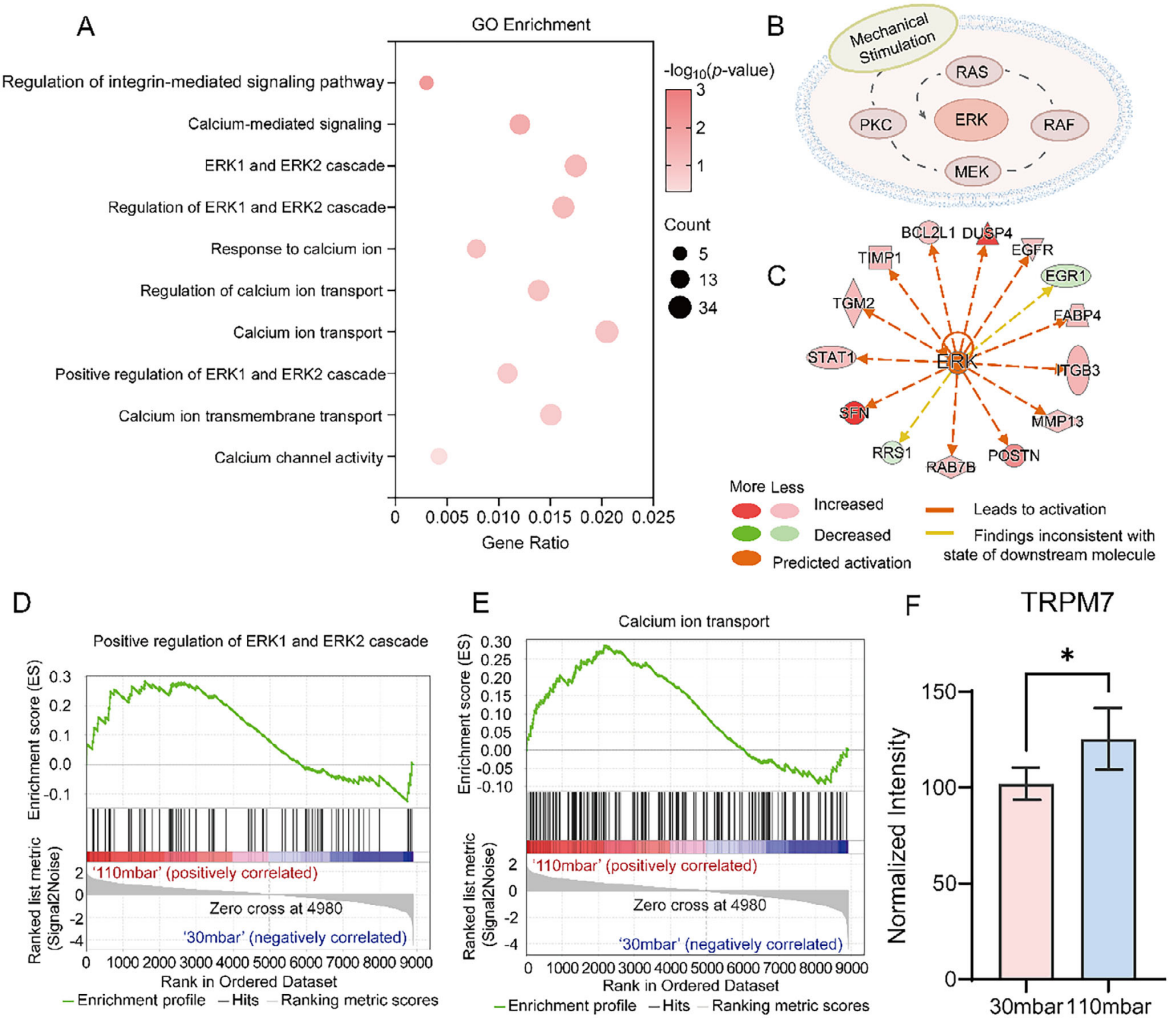
Proteomic analysis of MOVAS responses to mechanical cues under the pathological stretch. A) Pathways related to cellular responses to mechanical stress from GO enrichment analysis of the significantly changed proteins between pathological and physiological stretched groups. B) The PKC/ERK signaling pathway. C) IPA upstream regulator analysis predicted ERK activation. Dashed lines indicate an indirect relationship. D,E) GSEA predicted the activation of ERK1 and ERK2 cascade, as well as calcium ion transport. F) Upregulation of TRPM7 in the pathological stretched group. Data presented as mean±standard deviation, n = 4, *p*‐values were calculated using two‐tailed *t*‐test. ^*^ *p* < 0.05.

KEGG enrichment analysis suggested that cells respond to mechanical cues during pathological stretch. To further validate these findings, we performed GO enrichment on the significantly changed proteins. Terms related to integrin, calcium, and extracellular signal‐regulated kinase (ERK)—previously known to be linked to mechanical stimuli^[^ ^37^, ^38^, ^39^ ^]^—were detected. Both our in vitro vessel chip model and the in vivo Ang II‐treated mice model showed enrichment of these terms (**Figure** **4**A; Figure S6, Supporting Information). Multiple proteins involved in ERK signaling were dysregulated (Figure 4B). ERK signaling regulates fundamental cellular processes in response to extracellular cues, including protein synthesis, differentiation, cell‐cycle entry, cell survival, and cell motility.^[^ ^37^, ^40^, ^41^ ^]^ It mediates phosphorylation of ERK1/2, promoting transcription of genes associated with growth and mitogenic signals. Key mediators, such as Protein kinase C (PKC) and ERK1/2, play essential roles in responding to mechanical stress through intricate signaling networks. For instance, PKCε forms subcellular‐targeted signaling complexes with ERKs, wherein the mitochondrial PKCε‐ERK module mediates cardioprotective effects by phosphorylating and inactivating Bad.^[^ ^42^ ^]^ Shear stress also activates ERK1/2 in endothelial cells, influencing VSMC proliferation and migration via endothelial cell‐dependent mechanisms.^[^ ^43^ ^]^ IPA upstream regulator analysis predicted ERK activation in our in vitro model (Figure 4C), while gene set enrichment analysis (GSEA) indicated positive regulation of the ERK1 and ERK2 cascades under pathological stretch (Figure 4D). Proteins involved in the ERK signaling pathway exhibited an upregulation trend in the pathological stretch group (Figure S7, Supporting Information). Consistent with these findings, both GSEA and IPA confirmed ERK pathway activation in the in vivo mice model (Figure S8A,B, Supporting Information). Collectively, these results demonstrate that ERK signaling was activated under pathological stretch and in Ang II‐treated mice, aligning with established knowledge of hypertension pathogenesis.^[^ ^44^, ^45^ ^]^

The calcium ion transport pathway was also activated under pathological stretch and Ang II‐induced hypertension in mice, as indicated by GSEA enrichment (Figure 4E; Figure S8C, Supporting Information). Calcium ions serve as crucial second messengers that initiate and regulate VSMCs contraction.^[^ ^46^ ^]^ Excessive mechanical stretch can disrupt intracellular calcium homeostasis, leading to contractile dysfunction in VSMCs.^[^ ^47^, ^48^ ^]^ Inositol 1,4,5‐trisphosphate‐gated calcium channels (ITPRs), primarily located on the endoplasmic reticulum (ER) membrane, are pivotal for calcium signaling.^[^ ^38^ ^]^ Specifically, ITPRs bind inositol 1,4,5‐trisphosphate (IP3) and, upon activation, mediate the release of calcium ions into the cytoplasm. In our in vitro vessel chip model, ITPR1 and ITPR3 displayed an upregulation trend under pathological stretch, further suggesting intracellular calcium dysregulation (Figure S9, Supporting Information).

Transient receptor potential cation channel subfamily M member 7 (TRPM7), a mechanosensitive ion channel,^[^ ^49^ ^]^ is essential for maintaining and regulating intracellular calcium levels^[^ ^50^ ^]^ as well as mediating mechanical force sensing.^[^ ^51^ ^]^ Previous studies associate TRPM7 upregulation with loss of the contractile phenotype in VSMCs.^[^ ^50^ ^]^ Consistently, we observed elevated TRPM7 expression under pathological stretch (Figure 4F), indicating calcium dysregulation and a cellular response to mechanical cues. Integrins, which are involved in cell differentiation, proliferation, and survival, also contribute to mechano‐sensing.^[^ ^39^, ^52^ ^]^ In hypertension, integrin αV (ITGAV) expression is increased, driving vascular eutrophic inward remodeling.^[^ ^52^ ^]^ The enrichment of integrin signaling (Figure 4A) and the elevated trend of ITGAV (Figure S10, Supporting Information) likewise support a cellular response to mechanical stress in pathologically stretched MOVAS.

### 2.5 Proteomics Discloses Cellular Stresses Induced by Pathological Stretch

**Figure 5:**
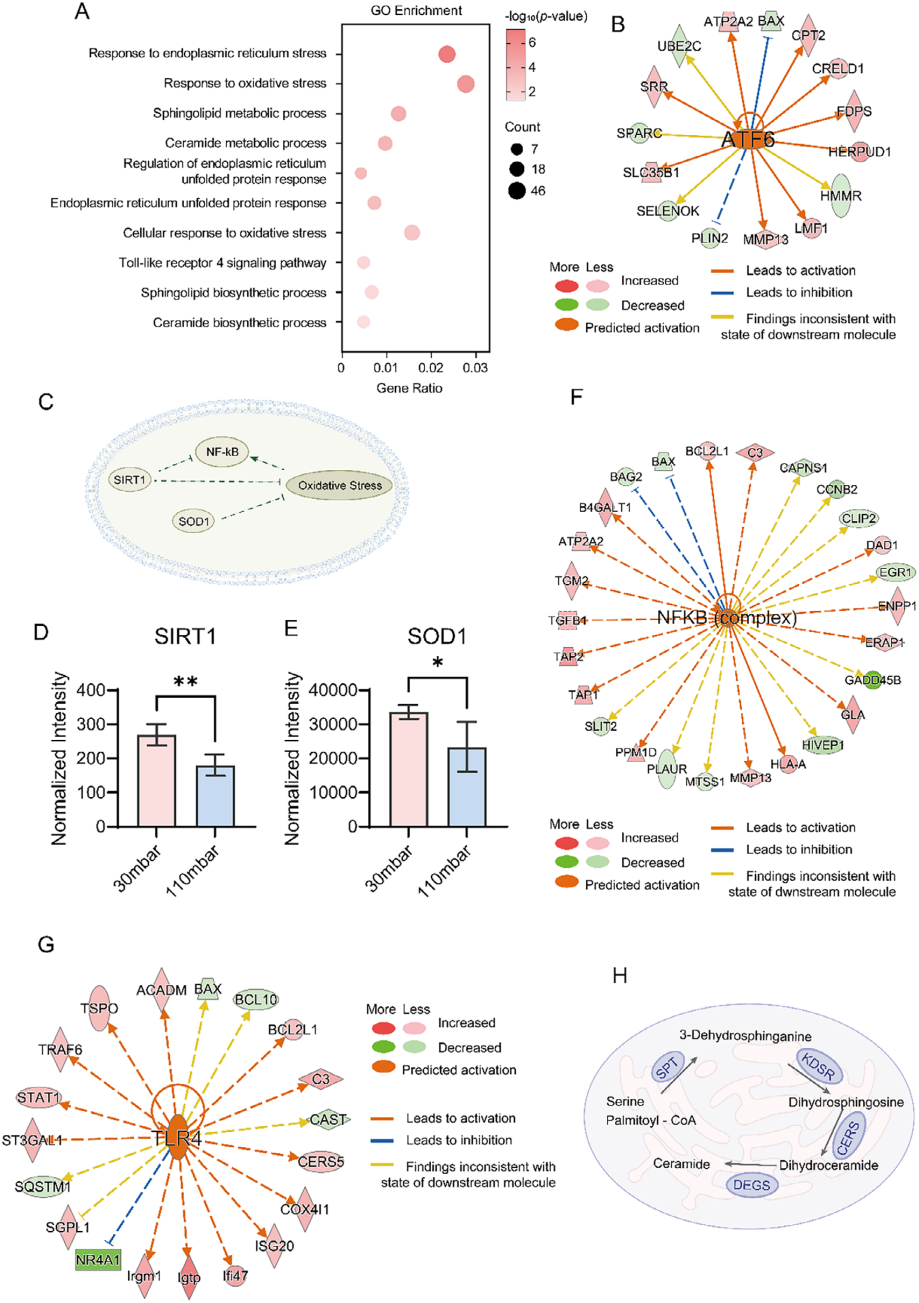
Proteomic characterization of MOVAS cellular stress responses under pathological stretch. A) Pathways related to cellular stresses identified through GO enrichment analysis of significantly altered proteins between the pathological and physiological stretched groups. B) ATF6 activation predicted by IPA upstream regulator analysis. Dashed lines indicate an indirect relationship, while solid lines indicate a direct relationship. C–E) Downregulation of SIRT1 (D) and SOD1 (E) in the pathological stretch group contributed to oxidative stress (data presented as mean±standard deviation, n = 4, *p*‐values calculated using two‐tailed *t*‐test. ^*^ *p* < 0.05, ^**^ *p* < 0.01). F) NF‐κB activation predicted by IPA upstream regulator analysis. G) TLR4 activation predicted by IPA upstream regulator analysis. H) Schematic representation of the ceramide biosynthesis pathway.

KEGG enrichment analysis also suggested cellular stress induced by pathological stretch. To further validate these findings, GO enrichment analysis was performed to investigate cellular stress responses in MOVAS cells and mouse vessels (**Figure** **5**A; Figure S11, Supporting Information). Under pathological stretch of cells or Ang II treatment of mice, multiple pathways were activated, particularly those related to ER stress, oxidative stress, pattern recognition receptors, and sphingolipid metabolism.

As the primary intracellular calcium reservoir, ER plays a fundamental role in protein maturation and folding. Impairment of its protein‐folding capacity leads to the accumulation of unfolded or misfolded proteins, triggering ER stress.^[^ ^53^ ^]^ This stress response is implicated in numerous CVDs, including hypertension. To mitigate ER stress, cells initiate the unfolded protein response (UPR), an adaptive mechanism aimed at restoring protein‐folding homeostasis and preserving cellular function. ATF6 is a crucial regulatory factor of the UPR. Under conditions of ER stress, ATF6 translocates from the ER to the Golgi apparatus, where proteolytic cleavage releases its active form. Once activated, ATF6 facilitates proper protein folding and drives the degradation of misfolded proteins, ultimately restoring ER function.^[^ ^54^, ^55^ ^]^ Our GSEA further revealed that ER stress and UPR pathways were activated in both the pathological stretch cell model and the Ang II‐treated mice model (Figure S12A,B and S13A,B, Supporting Information). Additionally, IPA upstream regulator analysis predicted the activation of ATF6 (Figure 5B; Figure S14A, Supporting Information), indicating that both MOVAS cells under pathological stretch and vessels in hypertensive mice respond to ER stress.

Oxidative stress, defined by an imbalance between the generation and elimination of reactive oxygen species (ROS), mediates cellular dysfunction, inflammatory responses, and apoptosis through mechanisms such as lipid peroxidation, protein oxidation, and DNA damage.^[^ ^56^ ^]^ It is a critical pathogenic factor in various diseases, including hypertension and other CVDs.^[^ ^33^ ^]^ Pathways related to oxidative stress were significantly enriched in both Ang II‐treated mice and MOVAS cells under pathological stretch (Figure 5A; Figure S11, Supporting Information). GSEA indicated hyperactivation of the cellular response to oxidative stress, suggesting a disruption of ROS homeostasis (Figures S12C and S13C, Supporting Information). Numerous proteins associated with oxidative stress were dysregulated in both models (Figure 5C). NAD‐dependent protein deacetylase sirtuin‐1 (SIRT1) is recognized for its protective role in various diseases, including vascular remodeling during hypertension and ROS regulation.^[^ ^57^, ^58^ ^]^ Our data showed that SIRT1 was downregulated under pathological stretch (Figure 5D), potentially exacerbating oxidative stress and contributing to the pathological phenotype. In the pathological stretch group, superoxide dismutase [Cu‐Zn] (SOD1) was downregulated (Figure 5E). SOD1 typically protects against oxidative stress by catalyzing the dismutation of superoxide anion (O_2_^•−^) into hydrogen peroxide (H_2_O_2_), which is subsequently broken down into water by catalase (CAT) and the glutathione pathway. Therefore, reduced SOD1 expression may lead to elevated ROS levels, exacerbating cellular oxidative stress.^[^ ^59^ ^]^ Additionally, CAT and glutathione pathway components (GLRX2, GPX4, GPX7) exhibited upregulating trends (Figure S15, Supporting Information), indicating the activation of compensatory antioxidant defense mechanisms.^[^ ^60^ ^]^

Our data also showed activation of the NF‐κB complex under pathological stretch (Figure 5F), a ROS‐sensitive transcription factor that plays a complex and pivotal role in oxidative stress and inflammatory responses.^[^ ^61^ ^]^ Excessive ROS production serves as a major trigger for NF‐κB activation. SIRT1 can inhibit the NF‐κB signaling pathway through multiple mechanisms,^[^ ^62^ ^]^ one of which involves enhancing antioxidant defenses—such as increasing the expression of superoxide dismutase (SOD)—to reduce ROS levels, and thereby indirectly suppress NF‐κB activation. Accordingly, the observed downregulation of SIRT1 likely contributed to NF‐κB activation in concert with increased ROS. Furthermore, endoplasmic reticulum oxidoreductase 1 (ERO1), which generates hydrogen peroxide in the ER,^[^ ^63^ ^]^ was upregulated in the pathologically stretched group, potentially exacerbating ROS burden and oxidative stress (Figure S16, Supporting Information). Consistently, analyses of vessels from hypertensive mice confirmed reduced SOD1 expression and SIRT1 inhibition, along with NF‐κB activation and ERO1 upregulation, aligning with our vessel chip model findings (Figure S14B–E, Supporting Information).

Toll‐like receptors 4 (TLR4), a key mediator of inflammatory responses, is associated with vascular remodeling and hypertension.^[^ ^64^ ^]^ When TLR4 is elevated, it can drive cell proliferation and inflammation, thereby promoting vascular pathology. Our results revealed the activation of TLR4 in both the hypertensive mouse model and the pathological stretch cell model (Figure 5G; Figures S12D, S13D, and S14F, Supporting Information), indicating that pattern recognition receptors mediate cellular stress responses under these conditions.

Sphingolipids are a class of complex lipids widely present in cell membranes, characterized by a sphingosine backbone that is amide‐linked to fatty acids to form ceramide, which in turn gives rise to a diverse array of sphingolipid species. In addition to serving as integral structural components of cell membranes, sphingolipids act as signaling molecules in various physiological and pathological processes, including apoptosis, inflammation, and vascular function.^[^ ^65^ ^]^ Previous studies have linked VSMCs injury and vascular remodeling to the synthesis or accumulation of ceramide and other sphingolipids.^[^ ^66^, ^67^ ^]^ Our results demonstrated that sphingolipid and ceramide biosynthetic pathways were significantly enriched in both the hypertensive mouse model and the pathological stretch cell model, as revealed by GSEA (Figure 5H; Figures S12E,F and S13E,F, Supporting Information). Moreover, proteins involved in ceramide synthesis displayed mainly upward trends in both pathological stretched cells and vessels from hypertensive mice (Figures S17 and S18, Supporting Information), suggesting that ceramide biosynthesis is activated under cellular stress, aligning with previous research.^[^ ^68^, ^69^ ^]^

### 2.6 Limitation Discussion of the Vessel‐On‐A‐Chip Platform

Our vessel chip model successfully recapitulated several cellular processes relevant to hypertension observed in vivo. Compared with traditional 2D cultures, a notable advantage of this vessel‐on‐a‐chip platform is its ability to closely mimic the native vascular microenvironment while enabling precise hypertension modeling through the modulation of cyclic mechanical strain. Nonetheless, several limitations remain. First, the vasculature comprises multiple cell types—including VSMCs, endothelial cells, monocytes/macrophages, and other immune cells—all of which function synergistically under physiological conditions. A single‐cell vessel chip model, lacking co‐culture with endothelial and immune cells, cannot fully capture the vascular dysfunction associated with hypertension. Second, in vivo hypertension triggers remodeling of ECM components (e.g., collagen, elastin), whereas microfluidic chips typically rely on a simplified ECM that fails to adequately reflect these changes. Moreover, mechanical cues can induce ECM reorganization in vessel chip model,^[^ ^21^, ^22^ ^]^ underscoring the need to investigate collagen remodeling for understanding hypertension pathophysiology. Advancements in biomaterial development—such as artificial hydrogels^[^ ^70^ ^]^ and inorganic biomaterials^[^ ^71^ ^]^—are therefore critical for more accurately reproducing the native ECM environment. Third, hypertension is a multifaceted pathological state involving intricate crosstalk among various organs and cell types, with immune regulation by the kidney and brain playing a particularly important role.^[^ ^72^ ^]^ Proteomic analysis of hypertensive mouse vessels revealed significant enrichment of pathways related to macrophage immune responses, highlighting the interplay between hypertension and immunology (Figure S19, Supporting Information). Nevertheless, our current vessel chip model lacks multi‐organ integration, precluding a comprehensive representation of these immune signals. Fourth, hypertension is a chronic condition, and short‐term microfluidic studies cannot adequately model the long‐term vascular remodeling it entails. Future developments should thus focus on developing more advanced models that better capture the in vivo immune interactions, the native ECM environment, the multi‐organ crosstalk, and the protracted remodeling processes associated with hypertension.

## 3 Conclusion

In summary, we developed a vessel‐on‐a‐chip model to simulate both healthy and hypertensive vascular conditions. A pressure of 30 mbar was applied to mimic physiological stretch, whereas 110 mbar simulated pathological stretch. Under physiological stretch, contractile marker expression was upregulated, reflecting a contractile phenotype and a healthier state in MOVAS cells. Conversely, pathological stretch resulted in the downregulation of these contractile markers, signifying a loss of contractile phenotype. Proteomic analysis demonstrated that MOVAS cells responded to mechanical cues and cellular stress under pathological stretch. To validate these findings, we established a mouse hypertension model using Ang II infusion. Observations from the vessel chip were consistent with the in vivo model, demonstrating comparable pressure‐overload‐induced remodeling of VSMCs. Notably, key pathways—including ERK signaling, calcium ion transport, integrin‐mediated signaling, ER stress response, oxidative stress response, TLR4 signaling, and ceramide synthesis—exhibited similar changes in the vessel‐on‐a‐chip system and the mouse hypertension model, signifying successful replication of in vivo conditions. This vessel‐on‐a‐chip system holds promise for advancing our understanding of vascular disease and facilitating the development of novel antihypertensive therapies. Future research could integrate multiple organs‐on‐a‐chip platforms to more accurately reflect the intricate cellular interactions underlying hypertensive pathology.

## 4 Experimental Section

### Microfluidic Device Design, Fabrication, and Assembly

The microfluidic device was adapted from a previously reported heart‐on‐a‐chip design.^[^ ^73^ ^]^ It consisted of an upper cell culture layer and a lower pneumatic layer, separated by a PDMS membrane. The upper channel was 3 mm in width and 170 µm in height and was divided into three channels by two columns of trapezoidal pillars. Each pillar measures 160 µm, with a 40 µm gap between pillars within a column and a 1 mm distance between the two columns. The master molds were fabricated using standard photolithography with SU‐8 2050 and SU‐8 2075 photoresist (MicroChem, USA) for the upper and lower channels, respectively, on a 3 inch silicon wafer substrate (Figure S20, Supporting Information). A direct write optical lithography system (Durham Magneto Optics Ltd., UK) was used to generate patterns. The PDMS pre‐polymer was mixed with a curing agent at a 10:1 weight ratio, degassed, and then poured into the master mold. Polymerization was carried out at 95 °C for 2 h.

The PDMS stamps were peeled from the molds after curing. Inlets and outlets for cells and culture medium were created using a steel needle with an outer diameter (OD) of 1.2 mm and inner diameter (ID) of 0.8 mm. The upper layer and the membrane were activated with oxygen plasma before assembly, followed by incubation at 95 °C for at least 2 h to ensure irreversible bonding. The pneumatic inlet and outlet were created with a needle of OD 0.8 mm and ID 0.6 mm and then the block was bonded with the pneumatic layer using oxygen plasma. Cells and culture medium were introduced into the microfluidic device using an 18‐gauge syringe with a steel needle (OD 1.2 mm, ID 0.8 mm) and polytetrafluoroethylene (PTFE) tubing (OD 2 mm, ID 1 mm). For the pneumatic layer, a 22‐gauge syringe with a steel needle (OD 0.8 mm, ID 0.6 mm) and PTFE tubing (OD 1.6 mm, ID 0.6 mm) were used. All syringes, needles, and tubing were washed with 75% ethanol and distilled water before being connected to the microfluidic chip with PDMS as a sealant.

### Cell Culture in the Microfluidic Device

The mouse aortic smooth muscle cell line MOVAS was obtained from Meisen CTCC (China). MOVAS cells were cultured in a DMEM medium (Solarbio, China) supplemented with 10% fetal bovine serum (FBS, ExCell, China), penicillin, and streptomycin. The cells were maintained and expanded in T25 cell culture flasks (Nest, China) until they were ready for use in the microfluidic device.

The microfluidic device was sterilized with 75% ethanol for 2 min and then washed twice with distilled water. After drying, the device was exposed to UV light for an additional 2 h for further sterilization. Before the cell culture, the cell culture channel was treated with dopamine hydrochloride (Sigma, USA) for 30 min and subsequently washed with distilled water. For cell seeding, MOVAS cells were digested and suspended in rat tail collagen type I (Corning, USA, 9.45 mg mL^−1^). The volume ratio of cell suspension to rat tail collagen to NaOH solution (0.13 mol L^−1^) was set at 2:1:0.15 to achieve a final concentration of 6 × 10^6^ cells mL^−1^. After thorough mixing, the cell suspension was injected into the chip. The device was then placed in a cell incubator for 15 min to facilitate the polymerization of the rat tail collagen. Subsequently, the two medium channels were connected to a syringe pump (LongerPump, China), and DMEM medium was injected at a flow rate of 1 µL min^−1^ to supply nutrients to the cells. After one day's static culture, the pneumatic channel was connected to a push‐pull pump (Fluigent, France) to provide cyclic pressure to the chip, applying pressures ranging from 0 to 30 mbar or 0 to 110 mbar at 1 Hz for 24 h, adapting from the previously described method.^[^ ^74^ ^]^ Statically cultured vessel chips were used as controls.

### Cyclic Strain Characterization

Cyclic strain characterization was conducted as previously described by tracking the displacement of polystyrene microspheres with diameters ranging from 5 to 5.9 µm (2.5% w/v, Aladdin, China) during stretching.^[^ ^75^ ^]^ The microspheres were first diluted in PBS (Solarbio, China) at a 1:15 ratio and then combined with rat tail collagen (9.45 mg mL^−1^, Corning, USA) in a 2:1 ratio, yielding a final concentration of 0.1% w/v. A concentrated NaOH solution (0.13 mol L^−1^) was added to adjust the pH to 7, facilitating collagen polymerization. This mixture was then injected into the middle channel of the upper layer and allowed to polymerize for 15 min. After polymerization, the side channels were filled with PBS.

Mechanical characterization was performed to deform the upper layer at a frequency of 1 Hz, with pressures varying between 0 and 30 mbar or 0 and 110 mbar. During cyclic stretching, microsphere movement was recorded using an inverted microscope (Cewei, China). The recorded frames were analyzed with ImageJ software,^[^ ^75^ ^]^ and a total of 150 microspheres from the left and right sides were randomly selected to calculate the deformation for each group.

Deformation along the x‐axis was calculated as follows:

(1) $$\mathrm{Disp}\mathrm{lace}\mathrm{ment}\;\mathrm{of}\;\mathrm{the}\;\mathrm{left}\;\mathrm{micr}\mathrm{osph}\mathrm{eres}=\;\;\frac{\Delta x}{x_{0}-x_{\max}}=\frac{x_{\max}-x_{\min}}{x_{0}-x_{\max}}\;$$

(2) $$Displacement\;of\;the\;right\;microspheres=\;\;\frac{\Delta x}{x_{min}-x_{0}}=\frac{x_{max}-x_{min}}{x_{min}-x_{0}}\;$$

where x_0_ represents the center position, and x_max_ and x_min_ denote the maximum and minimum x‐coordinates of the microspheres during stretching. The ratio of displacement along the y‐axis contributing to the total displacement was calculated as:

(3) $$Displacement\;contribution\;along\;y-axis=1-\;\frac{x_{max}-x_{min}}{\sqrt{{\left(y_{max}-y_{min}\right)}^{2}+{\left(x_{max}-x_{min}\right)}^{2}}}$$

where y_max_ and y_min_ represent the maximum and minimum y‐coordinates of the microspheres during stretching. The mean and standard deviation of deformation along the x‐axis were also calculated.

To evaluate the repeatability of the device, the average deformation of the collagen was measured under pressures of 0–30 mbar and 0–110 mbar for three chips separately. The mean and standard deviation of the deformation were calculated. Additionally, the deformation of the three chips under both pressure conditions was measured at 0 and 24 h after cyclic stretch to characterize the device's consistency.

### Immunofluorescence Staining

Immunofluorescence analysis was performed on the microfluidic chip for cell samples statically cultured and physiologically and pathologically stretched. Cell samples were sequentially incubated with 4% paraformaldehyde (Solarbio, China) for 10 min for fixation, 0.1% TritonX‐100 (Biosharp, China) for 8 min to permeabilize the cells, and 10% goat serum (Solarbio, China) for 90 min to block nonspecific binding. Following blocking, samples were incubated overnight with a rabbit anti‐MYH 11 antibody (1:100 dilution in PBST, Abcam, UK) in the presence of 2% goat serum. After washing with PBST, samples were further incubated with Alexa Fluor 488‐conjugated goat anti‐rabbit secondary antibody (1:250 dilution in PBST, ThermoFisher Scientific, USA) for 1 h. Fluorescence imaging was recorded using a Zeiss LSM 980 Inverted Confocal Microscope (Zeiss, Germany) with a 10x objective lens. The excitation wavelength was set at 488 nm, while the receiving wavelength was set as 490–551 nm. The excitation and max emission wavelengths of the secondary antibody were 499 and 520 nm. All the images were analyzed and quantified using ImageJ software and GraphPad Prism 10.

### In Vivo Assays for Hypertension Model

C57BL/6 mice (8–10 weeks old) were obtained from SiPeiFu Biotechnology Company (Beijing, China) and housed in groups of five in individually ventilated cages under controlled conditions (19–23 °C, 12 h light‐dark cycle). Mice were randomly divided into a treatment group and control group and anesthetized by intraperitoneal injection of a 4% chloral hydrate solution at a dose of 10 µL g^−1^ body weight. Following anesthesia, mini‐osmotic pumps (Model 1002, Alzet, USA) were implanted subcutaneously, with the catheter directed toward the mouse's head. Pumps were loaded with Ang II (Sigma–Aldrich, USA) diluted in saline according to the manufacturer's instructions. Each mouse received a continuous subcutaneous infusion of AngII (700 ng kg^−1^ per minute) or saline over a 14‐day period to induce hypertension.^[^ ^76^ ^]^ Mice were monitored daily, and blood pressure was measured pre‐surgery and on days 7 and 14 post‐surgery using the non‐invasive CODA blood pressure monitoring system (ADInstruments, New Zealand). After 14 days of Ang II infusion, mice were euthanized by CO₂ inhalation, and aortic tissue samples were collected for subsequent histological and proteomics analysis.

### Tissue Preparation and Paraffin Embedding

The vessels were fixed in 4% paraformaldehyde for 48 h, followed by running water rinsing for 6 h and three washes with distilled water, each lasting 3 min. Then the samples were dehydrated through a graded ethanol (Sinopharm, China) series and cleared using xylene (Sinopharm, China) as follows: 70% ethanol for 1 h; 80% ethanol for 1 h; 90% ethanol for 1 h; 95% ethanol for 2 h; anhydrous ethanol for 30 min (repeated twice); xylene for 8 min (repeated twice). For paraffin infiltration, the tissues were immersed in molten paraffin at 60 °C for 1 h, followed by two consecutive changes of fresh paraffin, each for 1 h. Finally, the paraffin‐infiltrated tissues were embedded in fresh paraffin using embedding molds. The paraffin blocks were allowed to cool and solidify, completing the preparation for subsequent sectioning and analysis.

### Masson's Trichrome Staining

Paraffin blocks were trimmed and sectioned at 5 µm thickness using a rotary microtome, ensuring complete vascular cross‐sections in each slide. Sections were floated on a 40 °C water bath, mounted onto clean glass slides, and dried in a 60 °C oven for 2 h to ensure adherence. Masson trichrome staining was performed using a commercial kit (Masson Staining Kit, Yifan Biotechnology, China). Slides were deparaffinized and rehydrated through the following sequence: xylene for 10 min (repeated twice); anhydrous ethanol for 5 min; 95% ethanol for 5 min; 80% ethanol for 5 min; 70% ethanol for 5 min, followed by rinsed with distilled water. For staining, sections were treated with Weigert's iron hematoxylin for 5 min to stain nuclei, rinsed with distilled water for 2 min, differentiated in 1% hydrochloric acid ethanol for a few seconds, and blued in distilled water for 5 min. Cytoplasm was stained using Ponceau‐acid fuchsin for 10 min, followed by a minute rinse in distilled water. Collagen fibers were differentiated with phosphomolybdic acid solution for 5 min, then stained with aniline blue for 5 min. Finally, sections were immersed in 1% acetic acid for 1 min to fix the staining. Dehydration and clearing were conducted through the following sequence: 70% ethanol for 2 min; 80% ethanol for 2 min, 95% ethanol for 2 min; anhydrous ethanol for 2 min; xylene for 5 min (repeated twice). Finally, the sections were mounted with neutral balsam. The sections were imaged using a digital slide scanner (JFBIO, China). All the images were analyzed and quantified using ImageJ software and GraphPad Prism 10.

### Proteins Extraction

The proteome samples were prepared using the EasyPept Micro Proteins Pre‐Processing and Preparation Kit (Omicsolution, China) as previously described.^[^ ^77^ ^]^ For the vessel chip model, after 48 h of incubation, the rat tail collagen was enzymolyzed by collagenases (Sigma, USA) diluted in TESCA buffer (Solarbio, China) at a concentration of 0.5 mg mL^−1^ until the cells were completely released from the collagen. Cells were collected and washed with PBS to remove the culture medium and then treated with the lysis buffer (reagent 0). The samples were lysed on ice for 10 min and then heated at 95 °C for 10 min followed by centrifuging at 12 000 rpm for 20 min at 4 °C to obtain the supernatants.

For vessel protein extraction, a lysis buffer composed of 1% sodium dodecyl sulfate (Sinopharm, China), 8M urea (Sigma–Aldrich, USA), and Halt Protease Inhibitor Cocktail EDTA‐free (ThermoFisher Scientific, USA) was added, followed by homogenization using a mechanical grinder (Jingxin, China). After grinding twice with 3 steel balls (−40 °C, 70Hz, on 120 s, off 180 s), the mixture was centrifuged at 12 000 rpm for 20 min at 4 °C to collect the supernatant. For each sample, 30 µg of protein was retrieved according to the quantification results using the Pierce BCA protein assay kit (ThermoFisher Scientific, USA). Acetone (Sinopharm, China) was added at a volume ratio of acetone:supernatant = 5:1 to precipitate protein at −20 °C overnight. Then, precipitation was washed with cold acetone 3 times and dried to remove acetone completely. 20 µL of reagent 0 of the EasyPept kit was added to each sample to dissolve protein, followed by centrifugation of the solution at 12 000 rpm for 20 min at 4 °C to obtain the supernatants.

Reagent A was added and heated at 95 °C for 5 min for protein denaturation and cysteine reduction/alkylation. After the solution was cooled, regent B containing trypsin was added to digest protein at 37 °C for 2 h and stopped by adding reagent C. The solution was centrifugated at 20 000 g for 1 min at 4 °C to obtain the supernatants. The supernatants were then desalinated through loading, washing, and elution. Pierce quantitative colorimetric peptide assay (ThermoFisher Scientific, USA) was used to quantify the peptides from mouse vessels, while a NanoDrop Microvolume Spectrophotometer (ThermoFisher Scientific, USA) was used to quantify the peptides from vessel chips. Peptides were collected and dried under a vacuum.

### LC‐MS/MS Analysis

Dried peptides were dissolved in water containing 0.1% formic acid to reach a final concentration of 200 ng µL^−1^. For analysis, 1 µL peptide solution was analyzed by a nanoElute liquid chromatography system (Bruker, Germany) coupled with a trapped ion‐mobility spectrometry quadrupole time‐of‐flight mass spectrometer (timsTOF Pro2, Bruker, Germany) using an AUR3‐15075 C18 column (150 mm × 75 µm, 1.7 µm, 120 Å pore size, IonOpticks, Fitzroy, Australia) at a column temperature of 60 °C. The mobile phases consisted of water (with 0.1% formic acid) as solvent A and acetonitrile (with 0.1% formic acid) as solvent B, at a flow rate of 300 nL min^−1^. The gradient elution profile was programmed as follows: 0 min at 2% B; 0–4 min, 2% to 3% B; 4–5 min, holding at 3% B; 5–46 min, 3% to 22% B; 46–52 min, 22% to 35% B; 52–57 min, 35% to 72% B; 57–60 min, holding at 72% B. The mass spectrometer operated in diaPASEF mode for data‐independent acquisition (DIA) to acquire mass spectrometry signal. Each analytical run was conducted over a total duration of 60 min, with precursor isolation windows set to 32 × 26 Th and the m/z scanning range from 400 to 1201 Da. During the MS/MS scanning, collision energy (CE) increased linearly from 20 eV (1/K_0_ = 0.6 Vs cm^−2^) to 59 eV (1/K_0_ = 1.6 Vs cm^−2^).

### Proteomics Data Analysis

Spectronaut 19 (Biognosys, Switzerland) was used to process and analyze the raw data through directDIA method with default settings. All the statistical analysis of the proteomic data was processed by R project versions 4.3, MetaboAnalyst 6.0 (https://www.metaboanalyst.ca/), ggplot2, and GraphPad Prism 10. For each protein, if the number of missing values exceeded 50% of the sample size, the protein was not included in the statistical analysis. Missing values were imputed with one‐fifth of the minimum identified value for the protein. Significantly changed proteins were defined as those with a FC greater than 1.5 or less than 2/3 as well as *p* < 0.05 (two‐tailed *t*‐test). The interaction and relation networks of significantly changed proteins were referred to the Kyoto Encyclopedia of Genes and Genomes (KEGG) pathway database (https://www.kegg.jp/) and Gene Ontology (GO) knowledgebase (https://geneontology.org/), with significance determined using hypergeometric testing. The prediction of pathway activation was performed using Gene Set Enrichment Analysis (GSEA) (https://www.gsea‐msigdb.org/gsea/index.jsp). Ingenuity Pathway Analysis (IPA, Qiagen, USA, https://digitalinsights.qiagen.com/IPA) was used to analyze and generate figures through upstream regulator analysis as well as diseases and functions analysis.

### Statistical Analysis

All in vitro experiments were performed at least in triplicate. Numerical data were presented as mean±standard deviation with a group number n described in the figure caption. Statistically significant differences between groups were assessed using a two‐tailed unpaired Student's *t*‐test, with significance defined as *p* < 0.05. All statistical analyses were carried out using GraphPad Prism 10.

### Ethics Statement

All experimental procedures were approved by the Animal Care and Use Committee of Shanghai Medical College, Fudan University (#DSF‐2020‐014).

### Data and Materials Availability

All proteome data have been deposited to the ProteomeXchange via the iProX partner^[^ ^78^, ^79^ ^]^ repository with the dataset identifiers PXD057724 and IPX0010186000.

## Conflict of Interest

The authors declare no conflict of interest.

## Author Contributions

Y.L. performed the experiments, analyzed the data, and wrote the first draft of the manuscript. J.Z. assisted with data processing. L.Z. and Z.W. assisted with protein extraction and immunofluorescence staining. D.Z. assisted with LC‐MS/MS analysis. H.L., X.Z., K.M., and X.Y. conducted the hypertensive mouse model and assisted with the related analysis. D.Z. assisted with photolithography. L.Q. and L.L. designed and supervised the study, provided funding, and finalized the manuscript.

## Supporting information



Supporting Information

Supporting Table 1‐6

Supplemental Video 1

Supplemental Video 2

## Data Availability

The data that support the findings of this study are openly available in ProteomeXchange at https://proteomecentral.proteomexchange.org/cgi/GetDataset?ID=PXD057724, reference number 57724.
