# Supplementary material for: Vessel‐On‐A‐Chip Coupled Proteomics Reveal Pressure‐Overload‐Induced Vascular Remodeling
Source: Adv Sci (Weinh). 2025 Mar 24;12(19):2415024. doi: 10.1002/advs.202415024 (PMC12097099; doi:10.1002/advs.202415024)
Supplement: Supplementary file 1 — Supporting Information [file ADVS-12-2415024-s004.pdf]

ADVANCED SCIENCE
Open Access

Supporting Information

for *Adv. Sci.*, DOI 10.1002/advs.202415024

Vessel-On-A-Chip Coupled Proteomics Reveal Pressure-Overload-Induced Vascular Remodeling

Yanjun Liu, Jianxujie Zheng, Lingyan Zhong, Zengyu Wang, Dan Zhao, Hong Lin, Xiaoxue Zhang, Ke Meng, Xiaoxia Yang, Dongxue Zhang, Ling Lin and Liang Qiao**

Supplementary information

Vessel-on-a-Chip Coupled Proteomics Reveal Pressure-Overload-Induced Vascular Remodeling

Yanjun Liu[1], Jianxujie Zheng[1], Lingyan Zhong[1], Zengyu Wang[1], Dan Zhao[1], Hong Lin[1], Xiaoxue Zhang[1], Ke Meng[2], Xiaoxia Yang[1], Dongxue Zhang[1], Ling Lin[1,*], and Liang Qiao[1,*]

1. Department of Chemistry, and Zhongshan Hospital, Fudan University, Shanghai 200000, China

2. Department of Neurosurgery, Zhongshan Hospital (Xiamen), Fudan University, Xiamen 361015, Fujian, China

Table of Contents

Figure S1. Comparison between collagen deformation at 0 and 24 hours. S4

Figure S2. Comparison of the MYL9, FHL2 and TPM1 between the physiological cyclic stretch group and the static culture group. S5

Figure S3 Heatmap clustering of the pathological and the physiological stretched cells based on proteomics data. S6

Figure S4 Construction of the hypertension mouse model. S7

Figure S5 Proteomic characterization of hypertensive mouse model. S8

Figure S6 Pathways related to cells response to mechanical stress in the *in vivo* hypertension mouse model. S9

Figure S7 The proteins involved in the ERK pathway between the physiological stretch group and the pathological stretch group. S10

Figure S8 Response to mechanical cues in the hypertension mouse model. S11

Figure S9 Comparison of ITPRs between physiologically and pathologically stretched groups. S12

Figure S10 Comparison of IGTAV between physiologically and pathologically stretched groups. S13

Figure S11 Pathways related to cellular stress responses in the hypertension mouse model. S14

Figure S12 GSEA analysis of cellular stresses induced by pathological stretch in MOVAS. S15

Figure S13 GSEA analysis of cellular stresses induced by Ang II treatment in mice. S16

Figure S14 IPA analysis and protein quantification for the Ang II-treated mouse model. S17

Figure S15 Comparison of the proteins associated with antioxidant defense between the pathologically and physiologically stretched groups. S19

Figure S16 Comparison of ERO1 between physiologically and pathologically stretched

groups. S20

Figure S17 Comparison of the proteins associated with ceramide synthesis between the pathologically and physiologically stretched groups. S21

Figure S18 Comparison of the proteins associated with ceramide synthesis between the Ang II-treated mice and the control group. S22

Figure S19 Immune responses in the *in vivo* hypertension mouse model. S23

Figure S20 The microfluidic device fabrication and assembly. S24

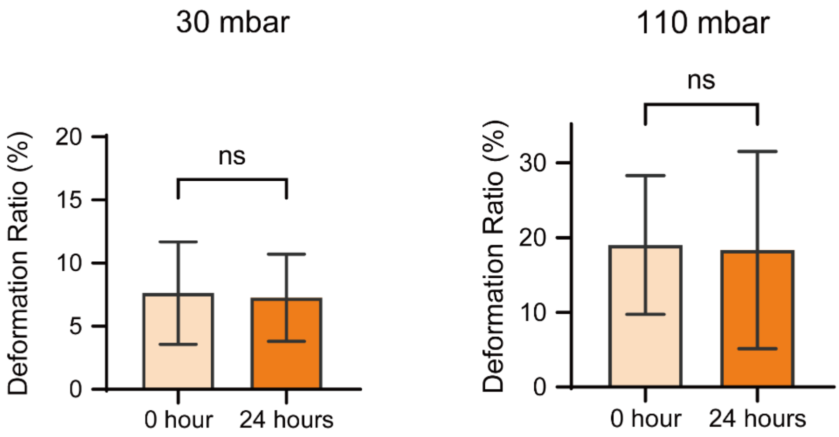

Figure S1. Comparison between collagen deformation at 0 and 24 hours of cyclic stretch under 30 mbar or 110 mbar.

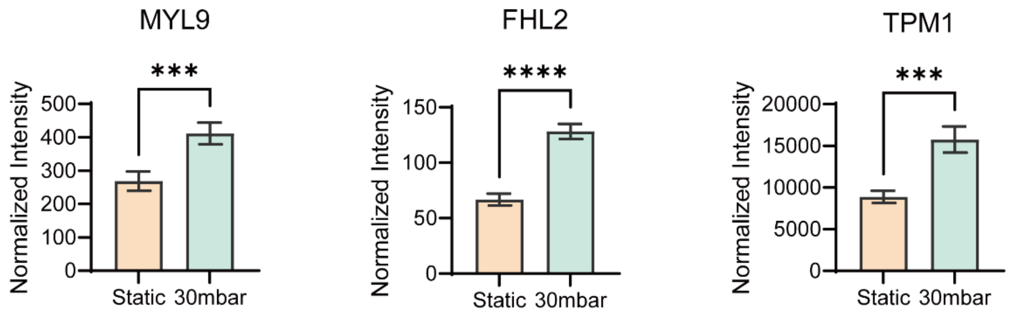

Figure S2. Comparison of the MYL9, FHL2 and TPM1 between the physiological cyclic stretch group and the static culture group. Data presented as mean±standard deviation, n=4, *p*-values were calculated using two tailed *t*-test. ***: $p < 0.001$, ****: $p < 0.0001$.

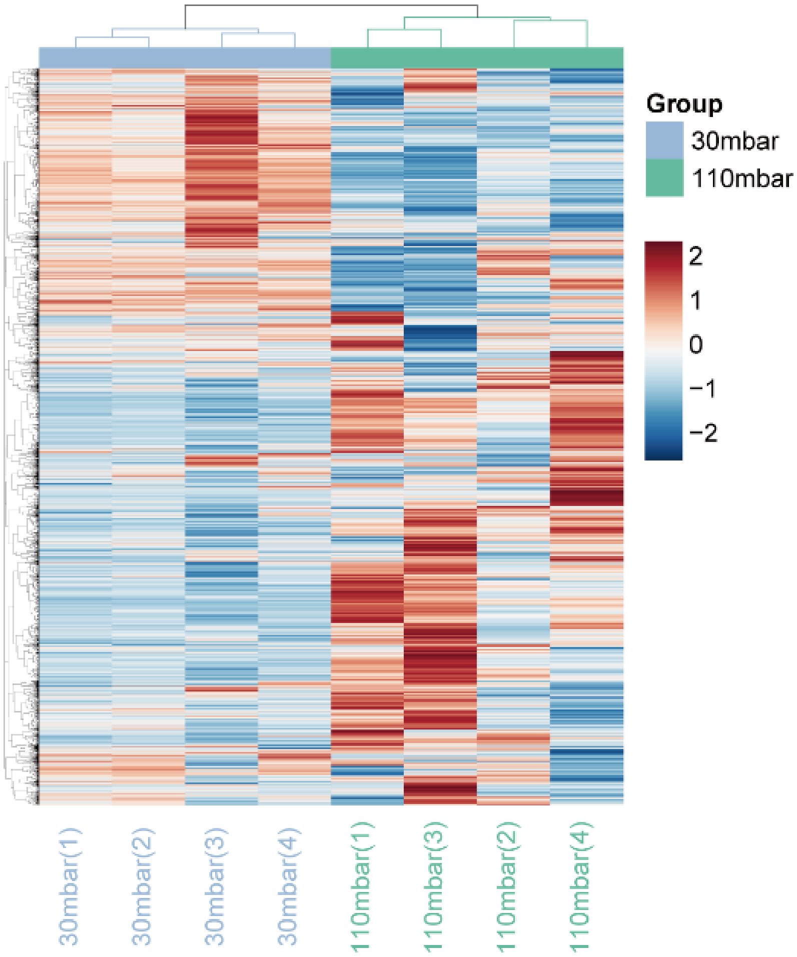

Figure S3. Heatmap clustering of the pathological and the physiological stretched cells based on the z-scores for protein contents. Pearson correlation was used as the distance measurement for both the columns and rows.

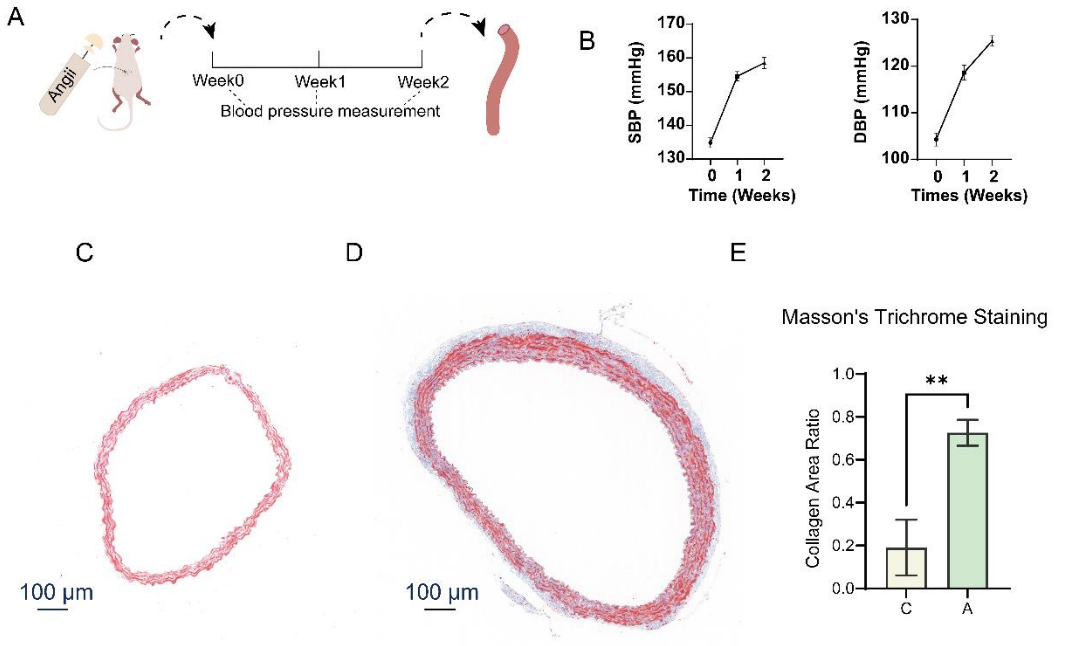

Figure S4. Construction of the hypertension mouse model. (**A**) The mouse hypertension model by Ang II treatment. (**B**) Blood pressure measurements were performed before Ang II treatment, as well as 7 days and 14 days after treatment. Six mice were included in each group. Representative images of Masson's trichrome staining in (**C**) the control group and (**D**) the Ang II-treated group. (**E**) Quantitative analysis of trichrome staining images showing the collagen area ratio between the Ang II-treated (A) and control (C) groups. Data presented as mean±standard deviation, n=3, *p*-value was calculated using two tailed *t*-test. ***: $p < 0.001$.

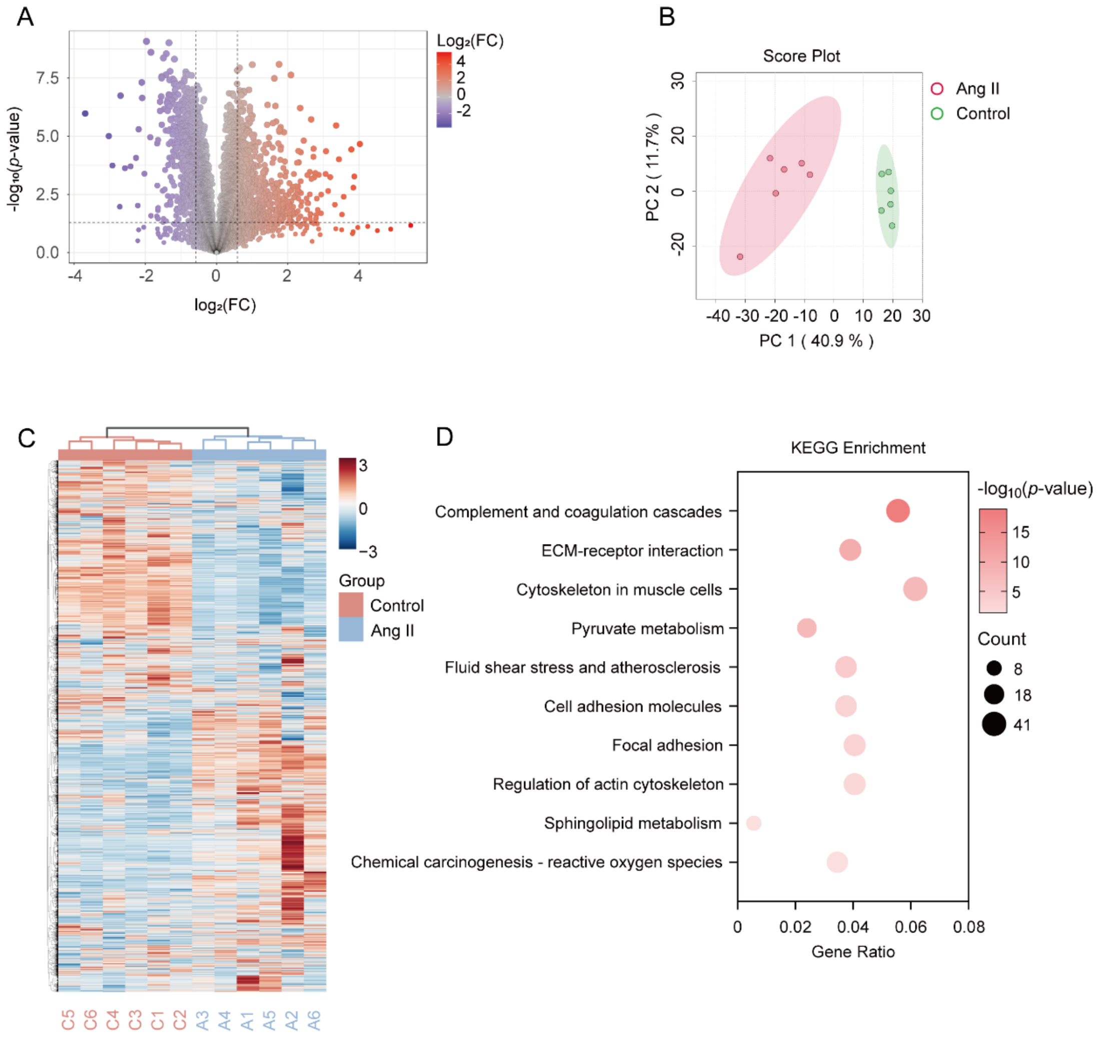

Figure S5. Proteomic analysis of the control and Ang II treated mice. (**A**) Volcano plots displaying identified proteins in vessels with $\log_2$FC as the horizontal axis and $-\log_{10}p$-value as the vertical axis. The thresholds were set as 0.05 for the *p*-value and 0.585 for $|\log_2\text{FC}|$. FC, fold change in protein expression of the Ang II-treated group compared to the wild group. (**B**) PCA score plots for proteomic data comparing Ang II-treated and wild mice groups. (**C**) Heatmap clustering of Ang II-treated and wild mice based on the z-scores for protein contents. Pearson correlation was used as the distance measurement for both the columns and rows. (**D**) Pathways from KEGG enrichment analysis of the altered proteins from (**A**).

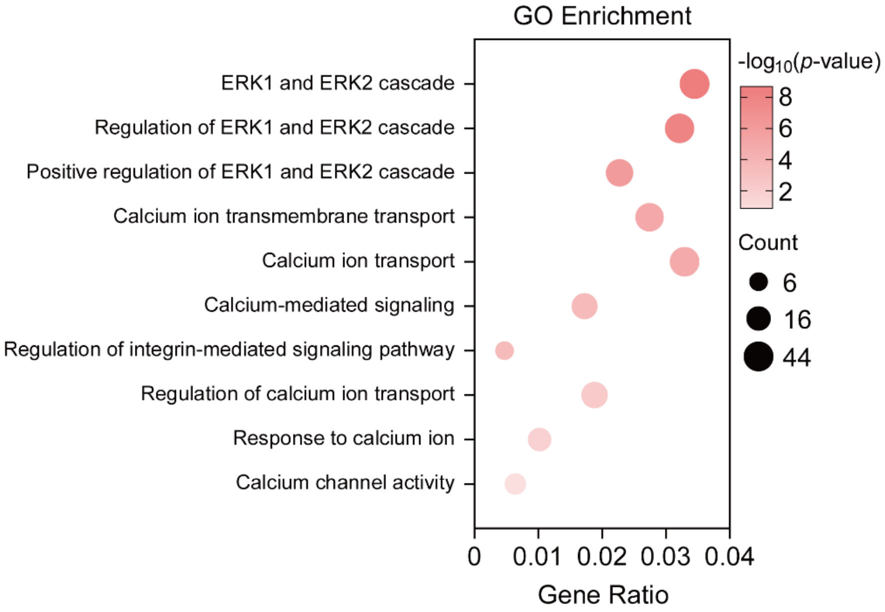

Figure S6. Pathways related to cells response to mechanical stress, from GO enrichment analysis of the proteins significantly altered (p-value <0.05 and $|log_2FC| > 0.585$) between the Ang II-treated and control mice groups.

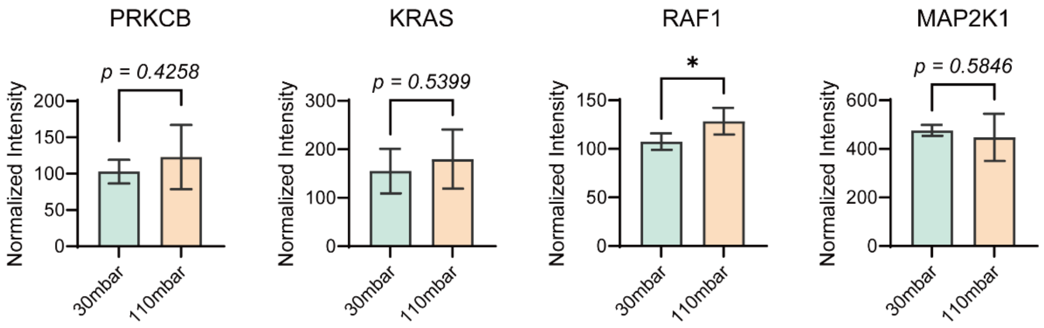

Figure S7. Comparison of the proteins involved in the ERK pathway between the physiological stretch group and the pathological stretch group. Data presented as mean±standard deviation, n=4, *p*-values were calculated using two tailed *t*-test. *: $p < 0.05$.

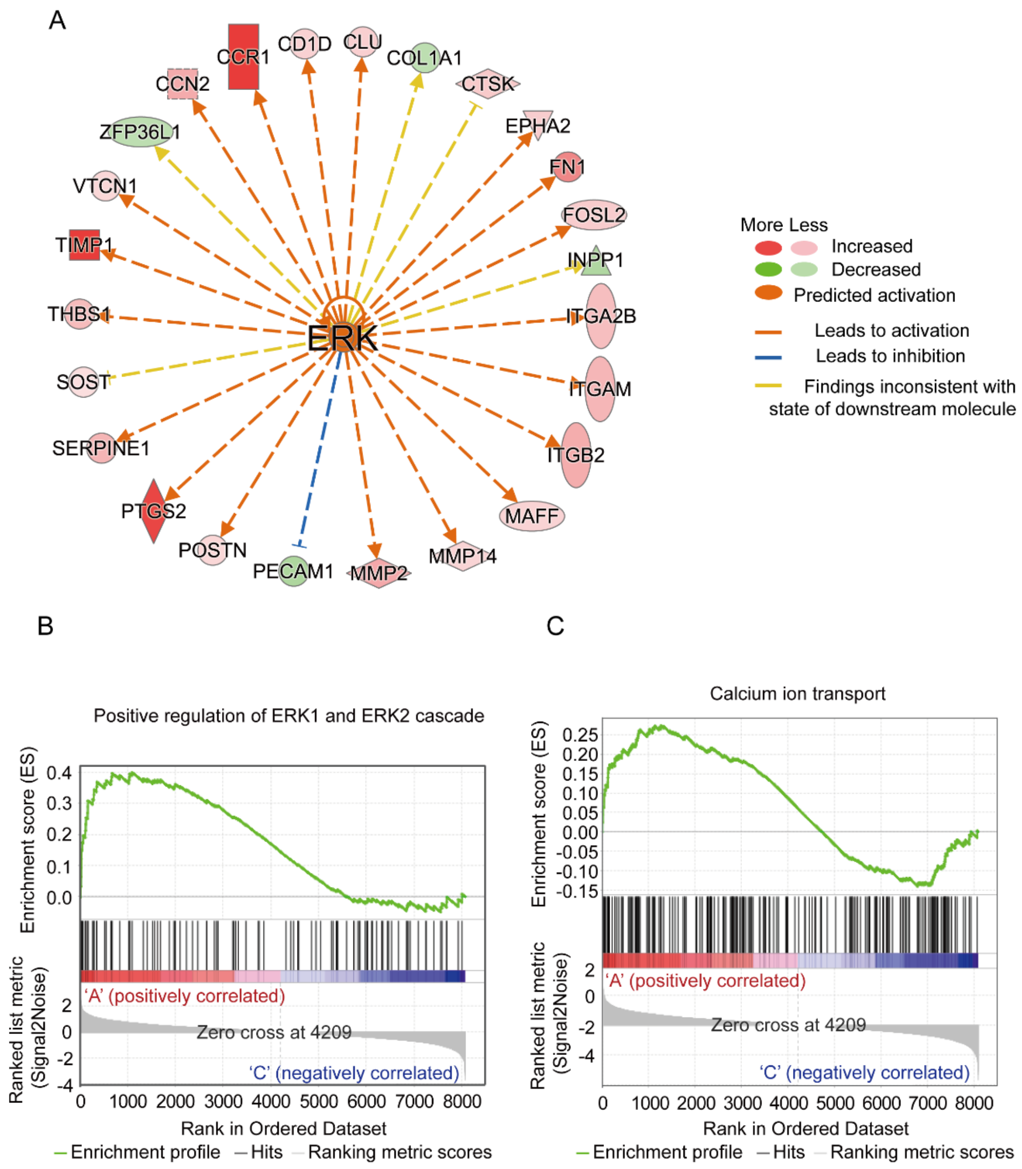

Figure S8. Response to mechanical cues in the *in vivo* hypertension mouse model. (**A**) The ERK genes were predicted to be activated by IPA upstream regulator analysis in the Ang II-treated mice. (**B**, **C**) GSEA was employed to predict the activation of positive regulation of ERK1 and ERK2 cascade as well as calcium ion transport in the Ang II-treated mice.

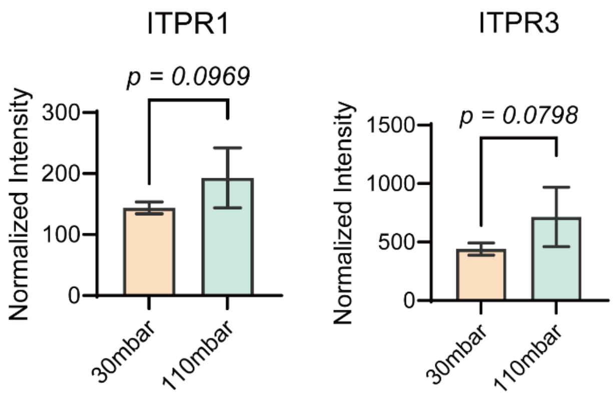

Figure S9. Comparison of ITPR1 and ITPR3 between physiologically and pathologically stretched groups. Data presented as mean±standard deviation, n=4, *p*-values were calculated using two tailed *t*-test.

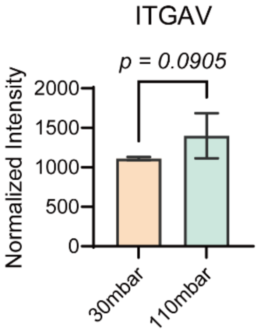

Figure S10. Comparison of IGTAV between physiologically and pathologically stretched groups. Data presented as mean±standard deviation, n=4, *p*-value was calculated using two tailed *t*-test.

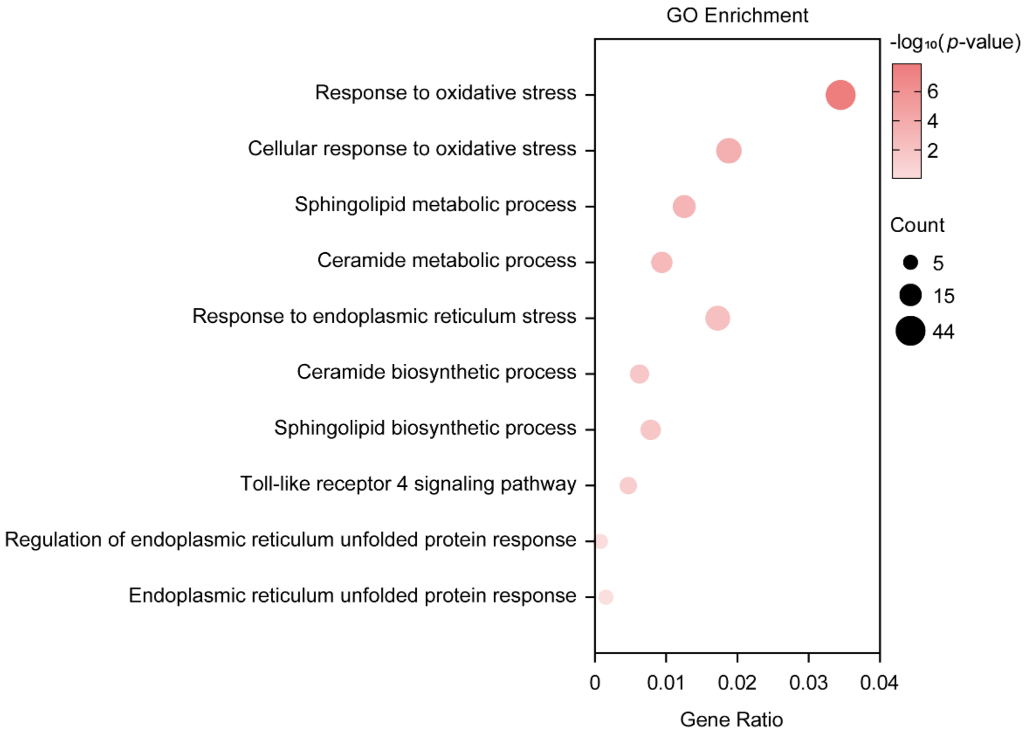

Figure S11. Pathways related to cellular stress responses, derived from GO enrichment analysis of the proteins significantly altered (p-value < 0.05 and $|log_2FC|$ > 0.585) between the Ang II-treated and wild type mice groups.

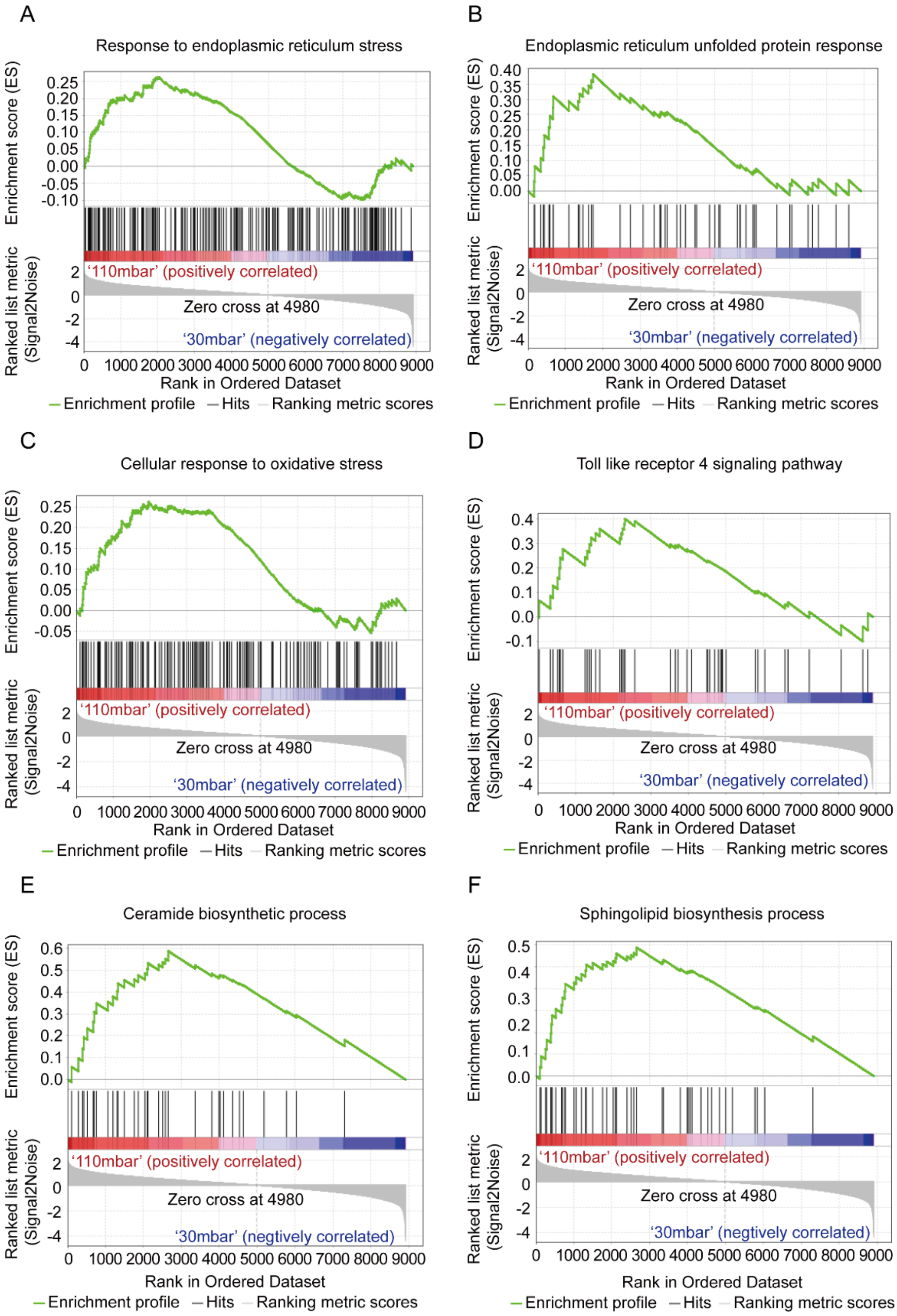

Figure S12. GSEA analysis of cellular stresses induced by pathological stretch in MOVAS. (**A**) Response to endoplasmic reticulum stress, (**B**) endoplasmic reticulum unfolded protein response, (**C**) cellular response to oxidative stress, (**D**) toll like receptor 4 signaling pathway, (**E**) ceramide biosynthetic process, (**F**) sphingolipid biosynthesis process.

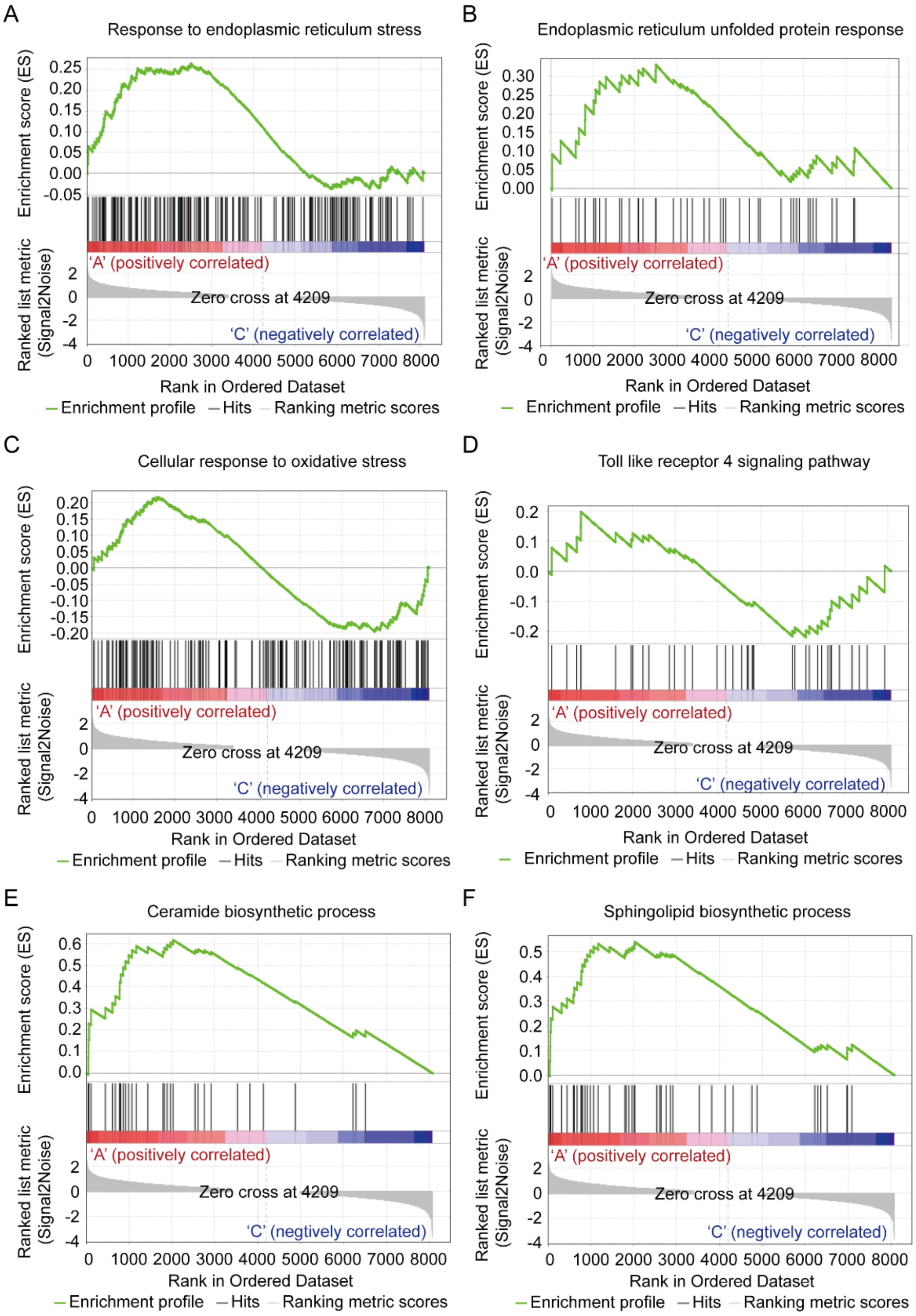

Figure S13. GSEA analysis of cellular stresses induced by Ang II treatment in mice. (**A**) response to endoplasmic reticulum stress, (**B**) endoplasmic reticulum unfolded protein response, (**C**) cellular response to oxidative stress, (**D**) toll like receptor 4 signaling pathway, (**E**) ceramide biosynthetic process, (**F**) sphingolipid biosynthesis process.

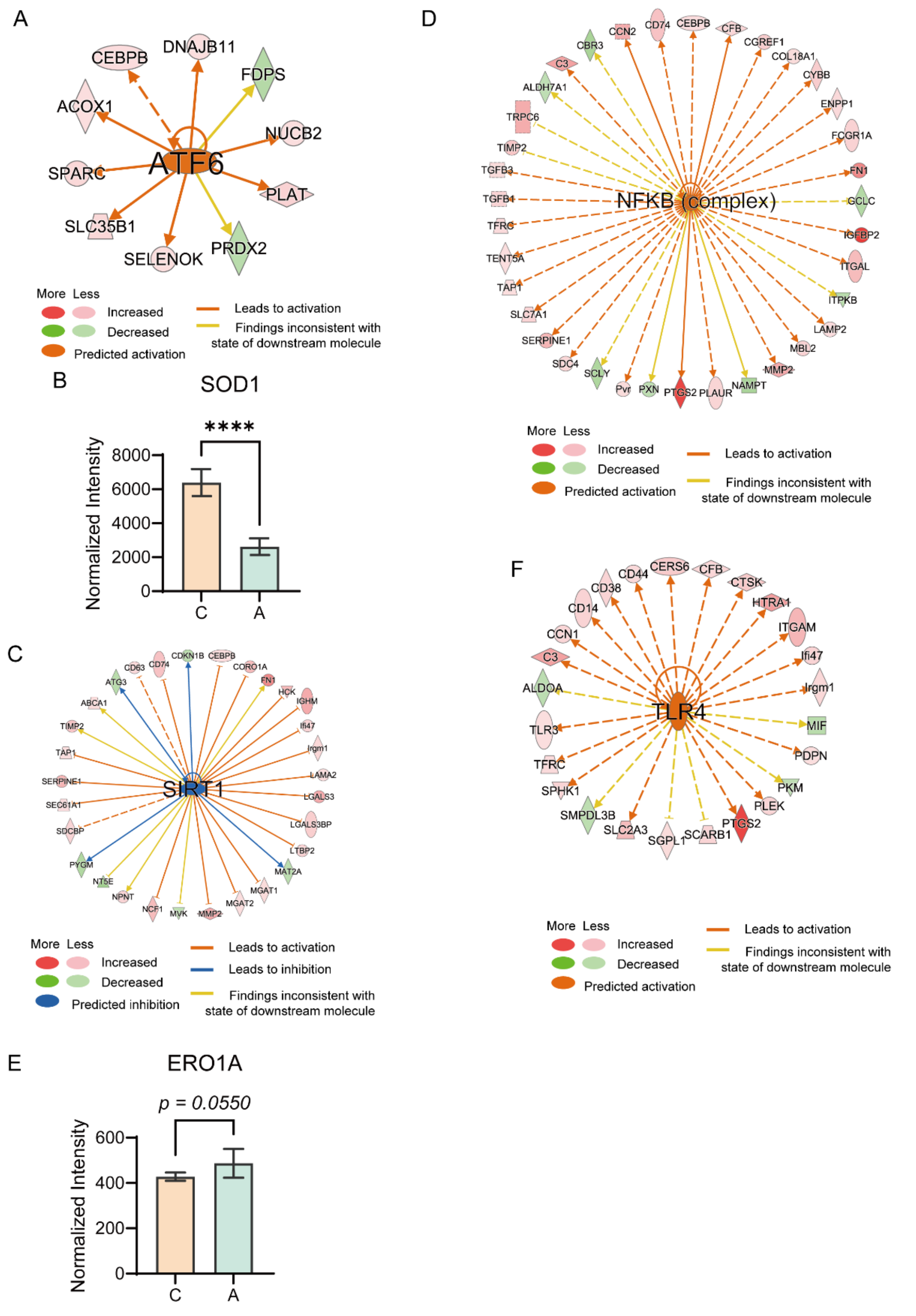

Figure S14. IPA analysis and protein quantification for the Ang II-treated mouse model. (**A**) The ATF6 was predicted to be activated in the Ang II-treated mice by IPA upstream regulator analysis. (**B**) Downregulation of SOD1 in the Ang II-treated mice. Data presented as mean±standard deviation, n=6, *p*-values were calculated using two

tailed *t*-test. ****: $p < 0.0001$. (**C**) The SIRT1 was predicted to be inhibited and (**D**) the NF-κB were predicted to be activated in the Ang II-treated mice by IPA upstream regulator analysis. (**E**) ERO1A exhibited upregulation trends in the Ang II-treated mice. Data presented as mean±standard deviation, n=6, *p*-value was calculated using two tailed t-test. (**F**) TLR4 were predicted to be activated in the Ang II-treated mice by IPA upstream regulator analysis.

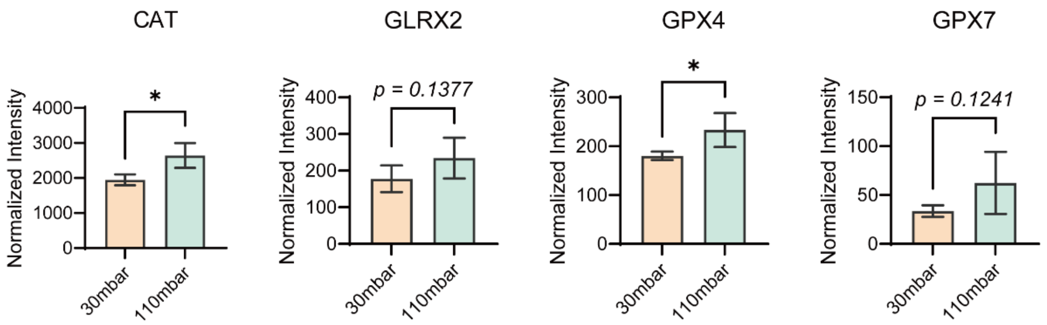

Figure S15. Comparison of the proteins associated with antioxidant defense between the pathological and physiological stretched groups. Data presented as mean±standard deviation, n=4, *p*-values were calculated using two tailed *t*-test. *: $p < 0.05$.

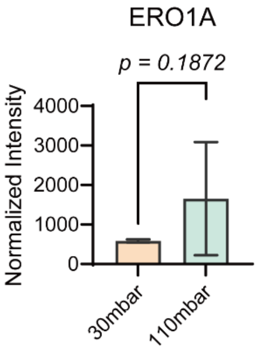

Figure S16. Comparison of ERO1A between physiologically and pathologically stretched groups. Data presented as mean±standard deviation, n=4, *p*-value was calculated using two tailed *t*-test.

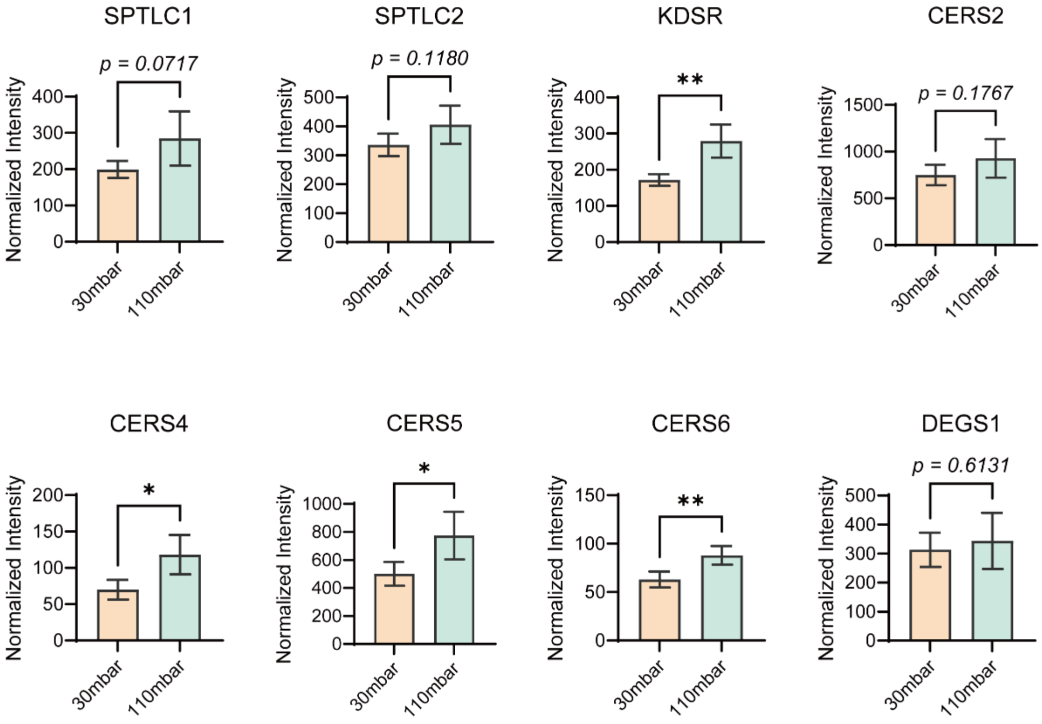

Figure S17. Comparison of the proteins associated with ceramide synthesis between the pathologically and physiologically stretched groups. Data presented as mean±standard deviation, n=4, *p*-values were calculated using two tailed *t*-test. *: $p < 0.05$, **: $p < 0.01$.

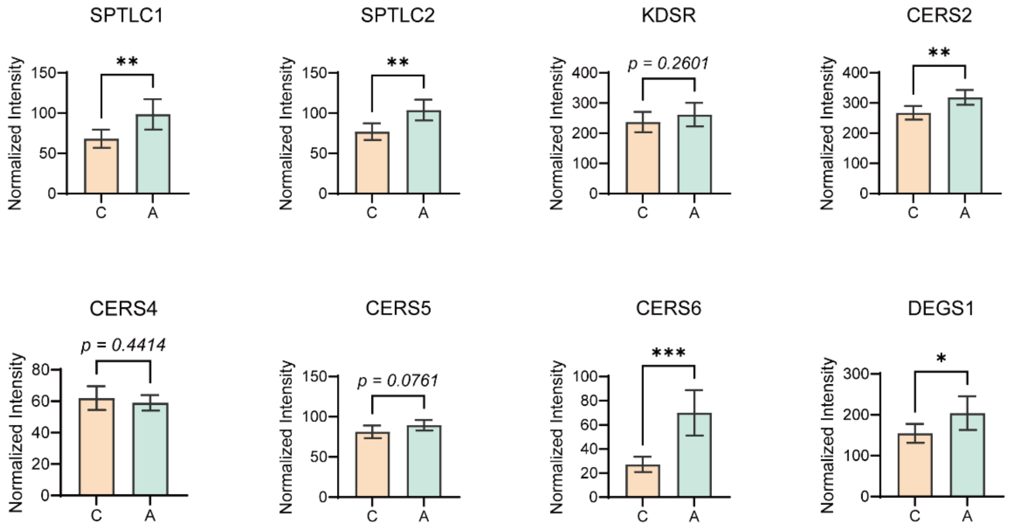

Figure S18. Comparison of the proteins associated with ceramide synthesis between the Ang II-treated mice and the control group. Data presented as mean±standard deviation, n=6, *p*-values were calculated using two tailed *t*-test. *: $p < 0.05$, **: $p < 0.01$, ***: $p < 0.001$.

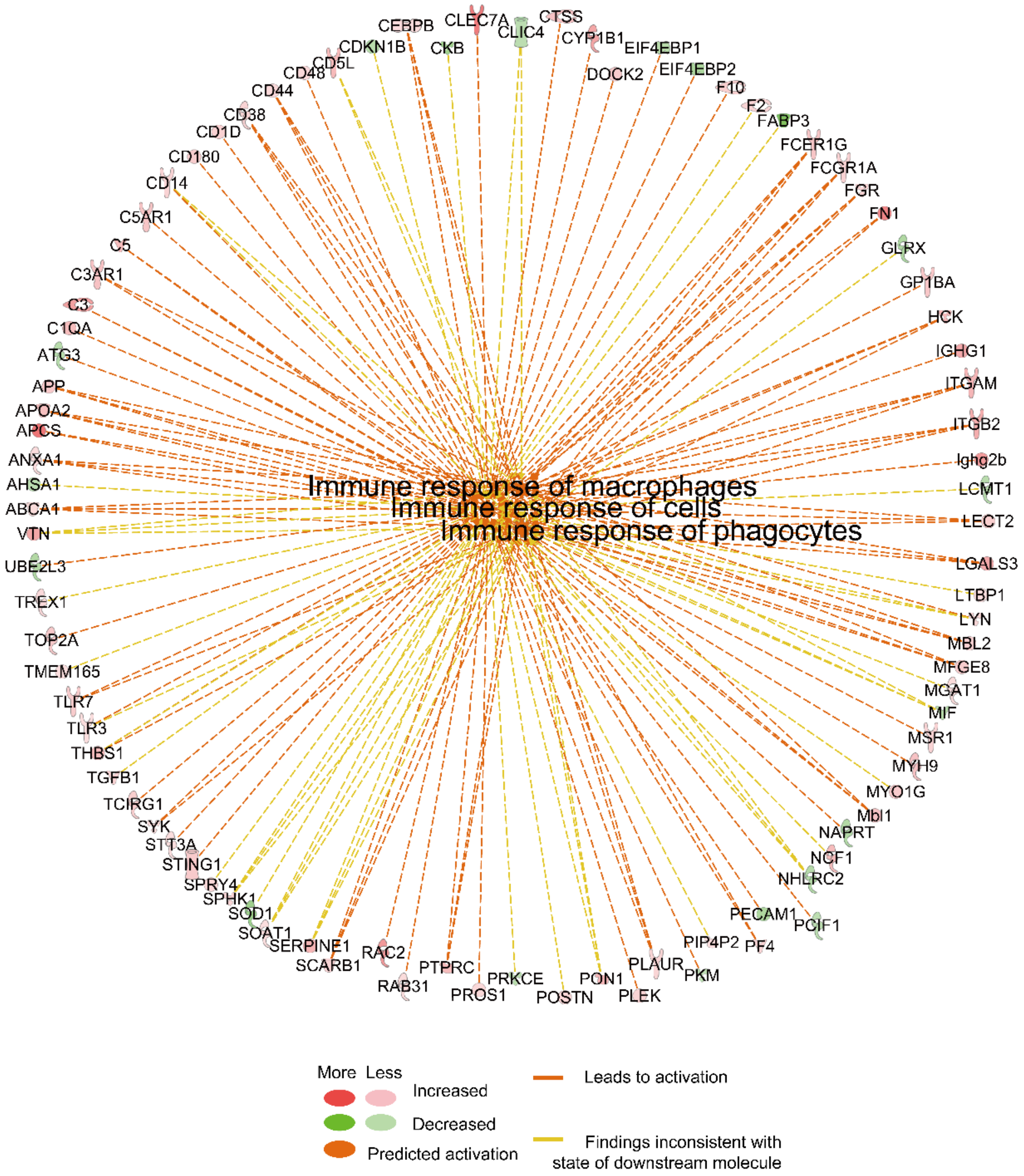

Figure S19. Activation of immune response of macrophages, immune response of cells and immune response of phagocytes predicted by IPA diseases and functions analysis for the Ang II-treated mouse model.

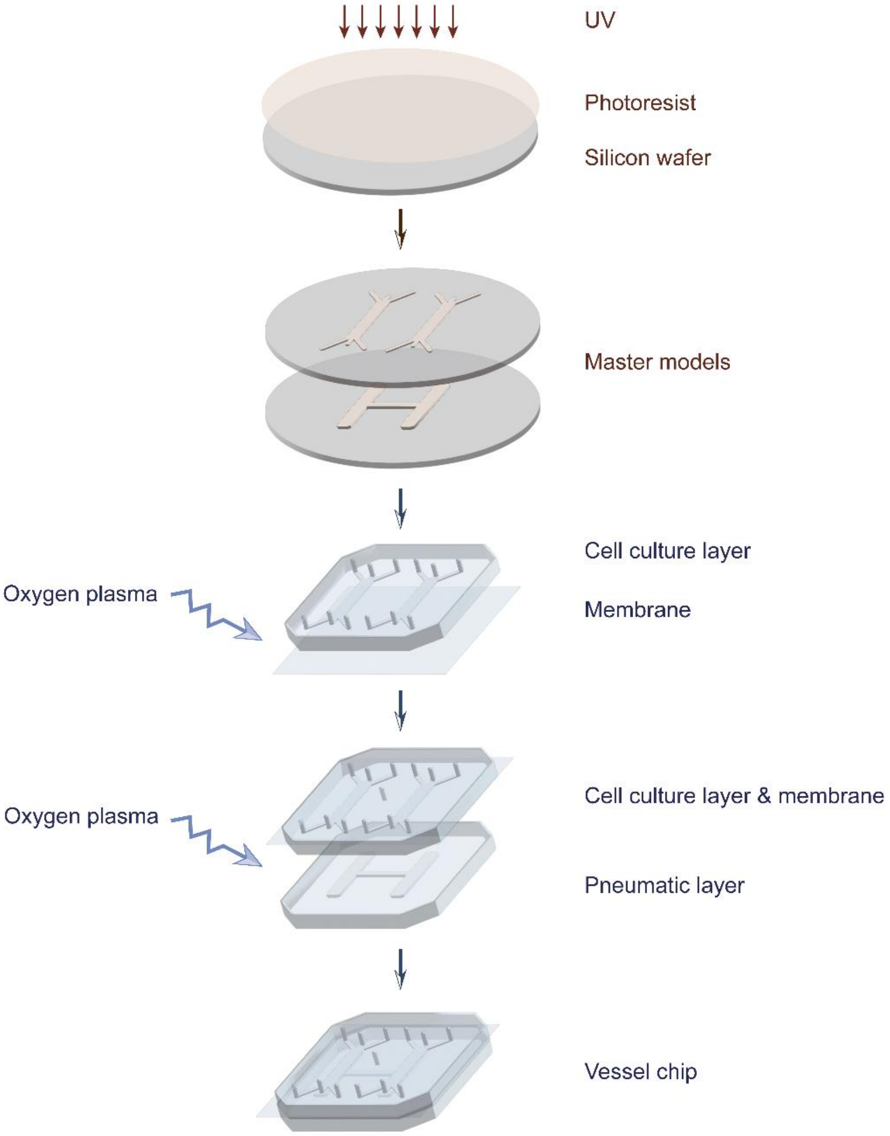

Figure S20. The microfluidic device fabrication and assembly. The master molds were fabricated using standard photolithography with SU-8 2050 and SU-8 2075 photoresist. The PDMS stamps were peeled off from the molds after curing, activated with oxygen plasma, and assembled layer-by-layer. On one device, there are two vessel chips sharing the connected pneumatic chambers. With this design, one push-pull pump can induce the cyclic stretch of two chips.
